# Differential effects of inosine monophosphate dehydrogenase (IMPDH/GuaB) inhibition in *Acinetobacter baumannii* and *Escherichia coli*

**DOI:** 10.1128/jb.00102-24

**Published:** 2024-09-05

**Authors:** Yutian Peng, John G. Moffat, Cory DuPai, Eric M. Kofoed, Elizabeth Skippington, Zora Modrusan, Susan L. Gloor, Kevin Clark, Yiming Xu, Shuxuan Li, Liuxi Chen, Xingrong Liu, Ping Wu, Seth F. Harris, Shumei Wang, Terry D. Crawford, Chun Sing Li, Zhiguo Liu, John Wai, Man-Wah Tan

**Affiliations:** 1 Department of Infectious Diseases, Genentech Inc., South San Francisco, California, USA; 2 Department of Biochemical and Cellular Pharmacology, Genentech Inc., South San Francisco, California, USA; 3 Department of Bioinformatics, Genentech Inc., South San Francisco, California, USA; 4 Department of Proteomic and Genomic Technologies, Genentech Inc., South San Francisco, California, USA; 5 Department of Drug Metabolism and Pharmacokinetics, Genentech Inc., South San Francisco, California, USA; 6 Department of Structural Biology, Genentech Inc., South San Francisco, California, USA; 7 Department of Discovery Chemistry, Genentech Inc., South San Francisco, California, USA; 8 WuXi AppTec Co., Ltd., Waigaoqiao Free Trade Zone, Shanghai, China; NCBI, NLM, National Institutes of Health, Bethesda, Maryland, USA

**Keywords:** inosine monophosphate dehydrogenase, GuaB, *Acinetobacter baumannii*, *Escherichia coli*, *de novo* guanine biosynthesis, antibacterial

## Abstract

Inosine 5′-monophosphate dehydrogenase (IMPDH), known as GuaB in bacteria, catalyzes the rate-limiting step in *de novo* guanine biosynthesis and is conserved from humans to bacteria. We developed a series of potent inhibitors that selectively target GuaB over its human homolog. Here, we show that these GuaB inhibitors are bactericidal, generate phenotypic signatures that are distinct from other antibiotics, and elicit different time-kill kinetics and regulatory responses in two important Gram-negative pathogens: *Acinetobacter baumannii* and *Escherichia coli*. Specifically, the GuaB inhibitor G6 rapidly kills *A. baumannii* but only kills *E. coli* after 24 h. After exposure to G6, the expression of genes involved in purine biosynthesis and stress responses change in opposite directions while siderophore biosynthesis is downregulated in both species. Our results suggest that different species respond to GuaB inhibition using distinct regulatory programs and possibly explain the different bactericidal kinetics upon GuaB inhibition. The comparison highlights opportunities for developing GuaB inhibitors as novel antibiotics.

**IMPORTANCE:**

*A. baumannii* is a priority bacterial pathogen for which development of new antibiotics is urgently needed due to the emergence of multidrug resistance. We recently developed a series of specific inhibitors against GuaB, a bacterial inosine 5′-monophosphate dehydrogenase, and achieved sub-micromolar minimum inhibitory concentrations against *A. baumannii*. GuaB catalyzes the rate-limiting step of *de novo* guanine biosynthesis and is highly conserved across bacterial pathogens. This study shows that inhibition of GuaB induced a bacterial morphological profile distinct from that of other classes of antibiotics, highlighting a novel mechanism of action. Moreover, our transcriptomic analysis showed that regulation of *de novo* purine biosynthesis and stress responses of *A. baumannii* upon GuaB inhibition differed significantly from that of *E. coli*.

## INTRODUCTION

*Acinetobacter baumannii* is a Gram-negative, opportunistic human pathogen that is responsible for approximately 2%–4% of hospital-acquired infections globally (1). Due to environmental persistence and rapidly emerging multidrug resistance, *A. baumannii* was categorized as the top priority for research and development of novel antibiotics by the WHO and CDC (2, 3). *Escherichia coli* strains comprise a large and diverse group of Gram-negative bacteria, most of which are harmless and can be found in the environment and human microflora. However, pathogenic *E. coli* acquires virulence attributes and can cause a broad spectrum of diseases, including diarrhea, urinary tract infections and sepsis (4).

Inosine 5′-monophosphate dehydrogenase (IMPDH) catalyzes the first committed step in the synthesis of guanine nucleobases by oxidation of inosine 5′-monophosphate (IMP) to xanthosine 5′-monophosphate (XMP), which is further converted into guanosine 5′-monophosphate (GMP) by GMP synthase (5). Thus, inhibiting IMPDH results in depletion of GTP and dGTP, the building blocks for RNA and DNA, respectively. IMPDH is an enzyme that is conserved from prokaryotes to eukaryotes, but structural characterization has revealed that eukaryotic IMPDHs are divergent from the bacterial enzymes in the active site (6–15). Mycophenolic acid (MPA), a fungal metabolite discovered more than a century ago, specifically inhibits IMPDH and was developed as an immunosuppressant for preventing organ rejection and autoimmune diseases based on the fact that rapidly expanding human immune cells rely on *de novo* biosynthesis for their supply of guanine nucleotides (16, 17). However, MPA has limited activity against the bacterial GuaB. Together, these results suggest the potential for the design of selective antibacterials targeting GuaB enzyme.

**Fig 1:**
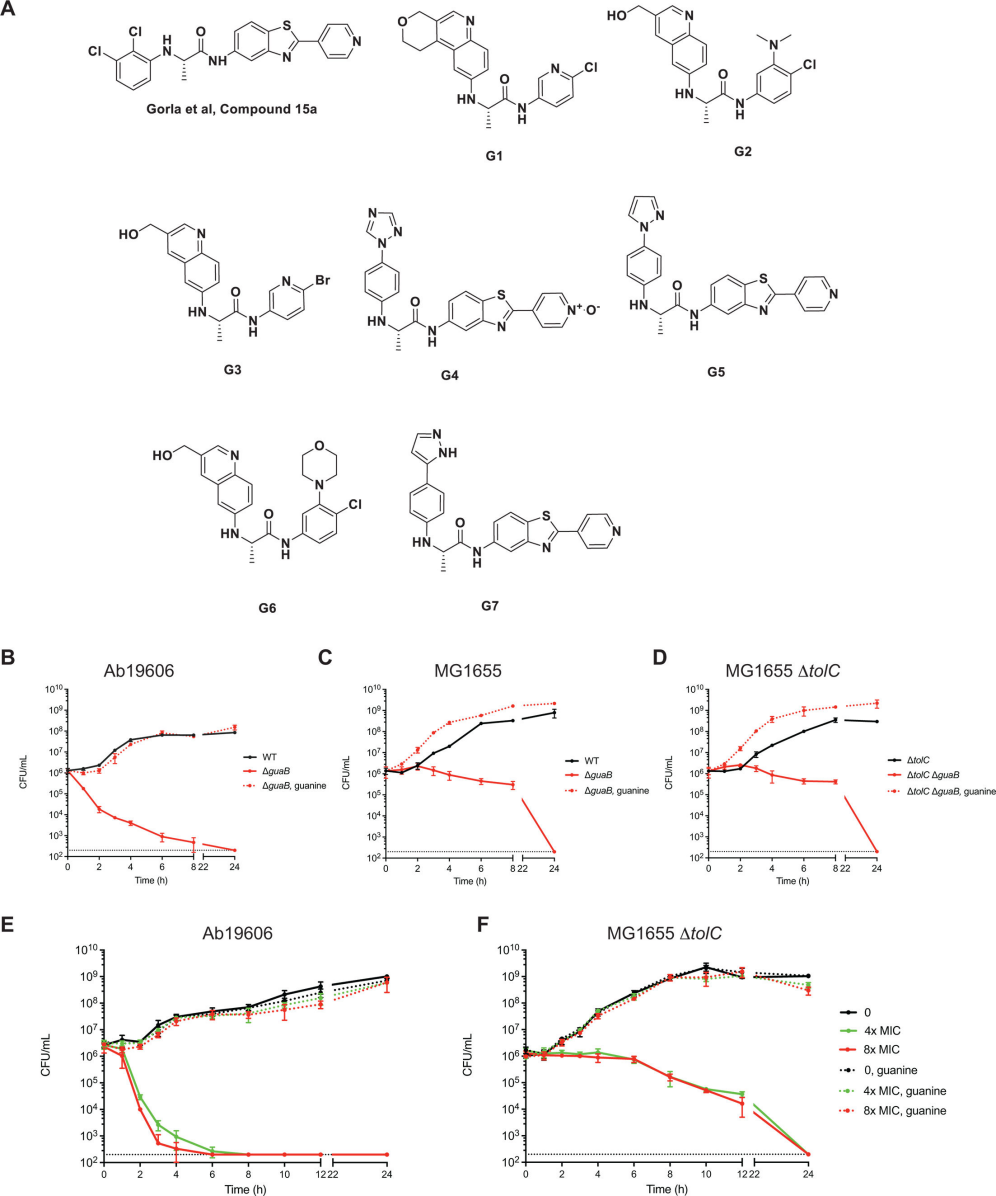
*A. baumannii* and *E. coli* exhibit distinct time-kill kinetics upon GuaB inhibition. (**A**) Chemical structures of published GuaB inhibitor 15a (26) and compounds G1–G7. (**B–D**) Time-kill assays of Ab19606 Δ*guaB,* MG1655 Δ*guaB*, and MG1655 Δ*tolC* Δ*guaB*. The *guaB*-null and corresponding wild-type strains were grown to log phase at 37°C in M9 media supplemented with 100 µM guanine and shifted to M9 media with or without guanine. Viable cells were serially diluted and plated on M9 agar plates supplemented with 100 µM guanine at the indicated time points. (**E and F**) Time-kill assays of Ab19606 and MG1655 Δ*tolC* with the GuaB inhibitor G6 at the concentration of 4× or 8× MIC. Viable cells were serially diluted and plated after exposure to G6 on M9 agar plates with or without 100 µM guanine. The averages and standard deviations of three independent experiments are shown. The dotted line indicates the minimum limit of detection, 200 CFU/mL.

In addition to *de novo* biosynthesis, many cells are able to obtain exogenous purines using nucleobase salvage pathways. A number of studies demonstrated that deletion of the *guaB* gene abolished growth *in vitro* under conditions in which the exogenous purines obtained through the salvage pathway are insufficient to compensate for GuaB deficiency. Such appears to be the case in infected tissues as *guaB* mutants of various bacterial pathogens are also avirulent (18–25), indicating salvage pathways alone are insufficient for bacterial viability *in vivo*. Beginning from previously published GuaB inhibitors (10, 26, 27), we developed a series of potent GuaB inhibitors (Fig. 1A) with 10^6^-fold selectivity for the *A. baumannii* GuaB enzyme over human enzymes. More importantly, these inhibitors achieve sub-micromolar minimum inhibitory concentrations (MICs) against the Gram-negative pathogen *A. baumannii* and the Gram-positive pathogen *Staphylococcus aureus* (Table 1).

**TABLE 1:** IC_50_ and MIC of GuaB inhibitors (μM)^*a*^

Compound	IC_50_				MIC				
	hIMPDH_2_	GuaB*^Ab^*	GuaB*^Ec^*	GuaB*^Sa^*	*Ab*	*Ab*Δ*lpxA*	*Ab*Δ*adeJ*	*Ec*Δ*tolC*	*Sa*
G1 (28)	>50	2.71 × 10^−4^	4.78 × 10^−1^	3.84 × 10^−4^	0.69	0.025	0.0159	>100	0.18
G2 (28)	>50	6.66 × 10^−4^	1.58 × 10^−2^	3.50 × 10^−3^	2	0.0695	0.0349	7.9	1.4
G3	>50	9.75 × 10^−4^	9.10 × 10^−2^	2.45 × 10^−3^	2.2	0.442	0.0552	59	1.6
G4	>50	6.17 × 10^−4^	9.03 × 10^−1^	6.20 × 10^−4^	2.2	0.156	0.039	>100	0.23
G5	>50	3.94 × 10^−4^	5.69 × 10^−1^	5.11 × 10^−4^	2.6	<0.1	<0.1	>100	0.39
G6	>50	5.64 × 10^−4^	5.90 × 10^−3^	8.01 × 10^−4^	3	0.078	0.0438	4	0.17
G7	>50	8.24 × 10^−4^	1.20	3.94 × 10^−4^	3.1	<0.1	<0.1	>100	0.17

^*a*^ Ab: ATCC19606; Ec: MG1655; Sa: USA300.

In this study, we examined the physiological and regulatory responses to GuaB inhibitors in *A. baumannii* and *E. coli*. We find that these inhibitors are bactericidal with a unique morphological signature in both *A. baumannii* and *E. coli* that differs from other antibiotics. Interestingly, the time-kill kinetics for *A. baumannii* and *E. coli* upon GuaB inhibition are distinct. Although GuaB inhibition results in drastic downregulation in siderophore biosynthesis and transport in both species, it induces opposite effects in regulation of the *de novo* purine biosynthesis and stress responses. These results reveal that inhibition of a highly conserved central metabolic pathway could lead to distinct responses in different species.

## RESULTS

### *A. baumannii* and *E. coli* exhibit distinct time-kill kinetics upon GuaB inhibition

GuaB is essential for Gram-negative bacterial viability in the absence of exogenous guanine (19, 21–24, 29–34). We constructed *guaB* deletions in an *A. baumannii* strain (Ab19606) (28), an *E. coli* strain (MG1655), and an efflux-defective *E. coli* mutant strain (MG1655 Δ*tolC*), and showed that these mutants are viable in M9 media only in the presence of guanine. Upon transfer to M9 media without guanine, the *guaB* mutant of *A. baumannii* 19606 died rapidly, with the colony forming units (CFUs) reduced by more than 3 logs in 6 h after removal of guanine (28). In contrast, the *guaB* deletion in wild-type *E. coli* (MG1655) or the efflux-defective mutant (MG1655 Δ*tolC*) exhibited less than a 1 log decrease in CFUs during the same time period (Fig. 1C and D). No viable bacteria was detectable 24 h after removal of guanine, and supplementation with guanine rescued the *guaB* knockout strains (Fig. 1B through D). The distinct death kinetics suggest that the loss of GuaB activity in the absence of guanine is bactericidal, and there are potentially distinct responses by different bacterial species.

To address the latter question, we used a series of GuaB inhibitors we developed to pharmacologically examine the differential responses of *A. baumannii* and *E. coli* to GuaB inhibition. These inhibitors are effective against *A. baumannii* with MICs ranging between 0.69 and 3.1 µM (Table 1). We found that the outer membrane barrier and efflux pumps are two major factors preventing the GuaB inhibitor from being effective against Gram-negative bacteria. For instance, a lipopolysaccharide (LPS)-deficient mutant Δ*lpxA* of *A. baumannii* had much lower MICs (ranging between 0.025 and 0.442 µM), and deletion of an efflux pump gene (*adeJ*) resulted in 26- to 68-fold decreased MICs. Similarly, deletion of *tolC*, which encodes the outer membrane subunit of multiple efflux pumps, rendered *E. coli* sensitive to a subset of the GuaB inhibitors (Table 1). Based on the fact that deletion of *guaB* in MG1655 Δ*tolC* had similar effects to the deletion in a wild-type MG1655 background (Fig. 1C and D), we concluded that TolC only affects efflux of the GuaB inhibitors and therefore used MG1655 Δ*tolC* in the rest of our study.

We chose to focus on inhibitor G6, which has comparable MICs against both Ab19606 and MG1655 Δ*tolC* (Table 1). To elucidate the binding mode of the G6 inhibitor, we determined the crystal structure of G6 bound to *A. baumannii* GuaB. The crystallographic asymmetric unit contains an octamer of GuaB, with each copy showing a clear density of G6 bound at the NAD + co-factor position adjacent to and contacting IMP, illustrating the direct interaction and plausible mechanism of inhibition (Fig. S1). This binding position allows G6 to interact with two neighboring protomers of GuaB, contacting several key residues (e.g., Glu416, Tyr445, and Ala249) that have been indicated as pivotal for species selectivity (8, 10). Similar to deletion of *guaB*, G6 had bactericidal effects in both species. At 4× the MIC, G6 was rapidly bactericidal against Ab19606, reducing CFUs by 3 logs within 6 h (Fig. 1E), whereas it decreased MG1655 Δ*tolC* CFUs by <1 log after 6 h (Fig. 1F). Similar to the *guaB* knockout strains, supplementing with guanine fully rescued GuaB inhibition (Fig. 1E and F). Thus, *A. baumannii* and *E. coli* exhibit distinct time-kill kinetics upon inhibition of GuaB. This difference in time-kill kinetics was unlikely due to the difference in IC_50_ of *A. baumannii* and *E. coli* enzymes because the *S. aureus* enzyme has a similar IC_50_ as the *A. baumannii* enzyme, yet we found that the time-kill kinetics of G6 in *S. aureus* were static over 24 h (Fig. S2). Similarities in the death kinetics between the *guaB* mutant (removal of GuaB protein by genetics) and GuaB inhibition (chemical inhibition of GuaB function), coupled with the co-crystal structure, support the specificity of G6 as a cellular GuaB inhibitor.

### GuaB inhibition leads to novel characteristic morphological changes

**Fig 2:**
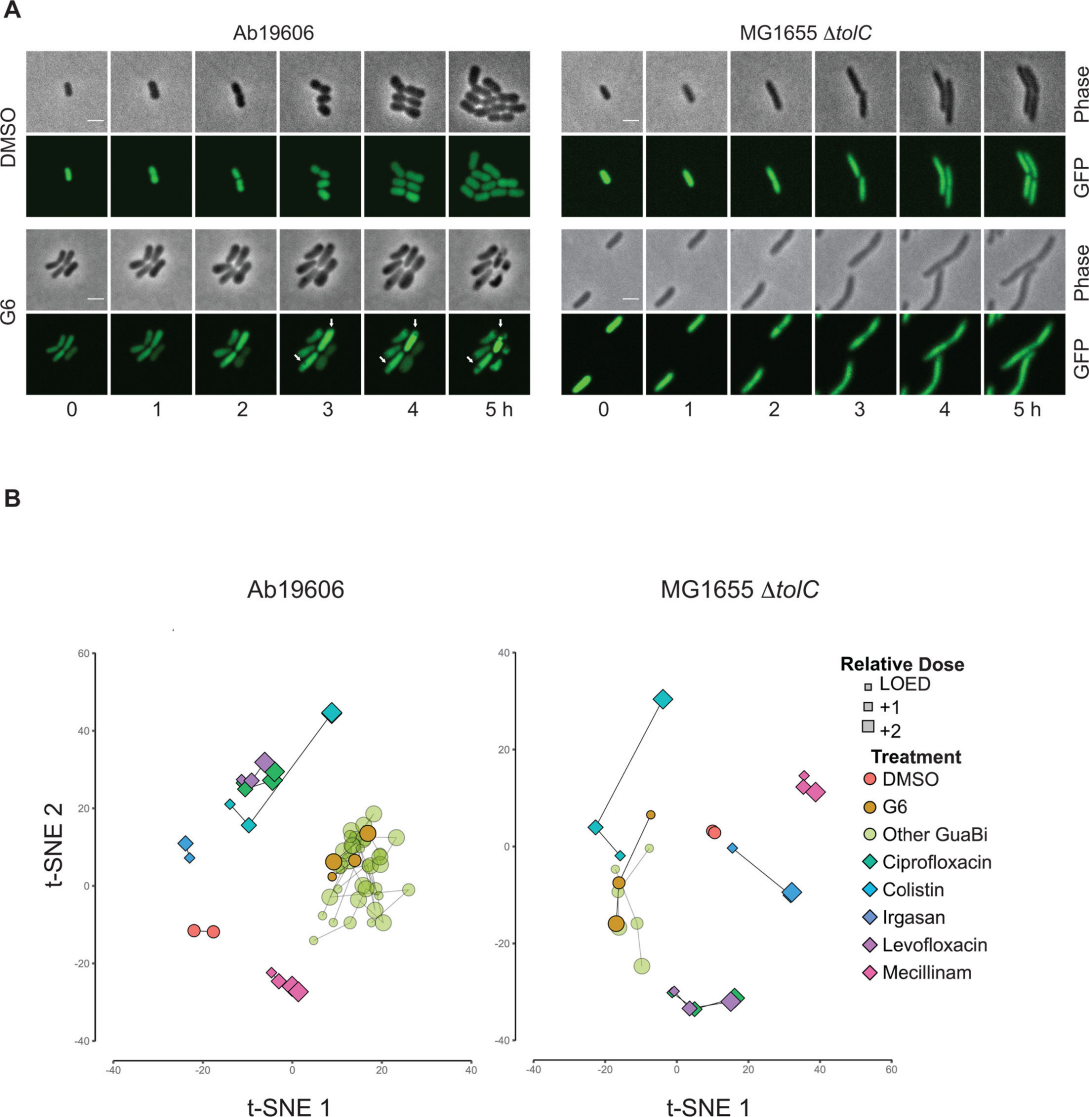
GuaB inhibition leads to novel morphological changes. (**A**) Morphology of Ab19606 and MG1655 Δ*tolC* bacterial cells treated with G6. The log-phase bacterial cells expressing cytoplasmic GFP were imaged at 37°C in M9 media containing G6 at 4× MIC. Representative images are shown. Scale bar, 2 µm. Arrows point at the cellular structures excluded from cytoplasmic GFP. (**B**) Phenotypic profiling of bacteria treated with reference antibiotics, G6, and other GuaB inhibitors (GuaBi). Multi-dimensional morphological and textural descriptors derived from microscopy images were used to cluster samples by t-SNE. Sizes of the symbols (“+1” and “+2”) indicate the relative dose in twofold increments compared with the lowest effective dose (LOED). Lines connect different doses of the same compound.

To understand the effects of GuaB inhibition in *A. baumannii* and *E. coli*, we imaged Ab19606 and MG1655 Δ*tolC* in the presence of 4× MIC of G6 for 5 h. Exogeneously expressed cytoplasmic GFP serves as an indicator for integrity of the bacterial cell envelope. As visualized by time-lapse microscopy (Fig. 2A), both strains grew in the absence of GuaB inhibitors. In the presence of G6, MG1655 Δ*tolC* cells continued to grow but failed to divide, resulting in elongated cells with evenly distributed GFP. Ab19606 cells also failed to divide, but instead of elongating, they became rounder. Moreover, cytoplasmic GFP was excluded from certain cellular structures that grew larger over time (Fig. 2A).

To quantify the observed morphological changes induced by GuaB inhibition, we applied an unbiased quantitative imaging approach. Previous reports using quantitative imaging showed that antibiotics with the same mechanism of action induce similar phenotypes, whereas different classes of antibiotics generate distinct morphological signatures (35, 36). Ab19606 and MG1655 Δ*tolC* were incubated with multiple concentrations of reference antibiotics or the GuaB inhibitors before staining with Sytox Green, which only stains cellular DNA upon outer membrane disruption, and FM4-64, which stains membranes. DNA was stained with DAPI after paraformaldehyde fixation. Images were processed to extract morphological feature descriptors. Following normalization, feature selection, and calculation of Euclidean distance from the DMSO control, the lowest observed effective dose (LOED) for each compound was determined as the lowest dose with a significant Euclidean distance from the DMSO control. Similarities and clustering of treatments were visualized using t-stochastic neighborhood embedding (t-SNE) clustering based on the selected morphological features. All GuaB inhibitors that possessed antibacterial activity in the MIC assay showed significant phenotypic effects at their respective LOEDs. All active GuaB inhibitors showed dose–response increases in phenotypic signatures and clustered together in the t-SNE plots for both Ab19606 and MG1655 Δ*tolC* (Fig. 2B). This strongly indicates that all GuaB inhibitors are acting by the same mechanism of action. Importantly, as seen with t-SNE clustering, each of the reference antibiotics with a distinct mechanism of action was differentiated from the others and from the GuaB inhibitors, whereas antibiotics with the same mechanism of action (e.g., ciprofloxacin and levofloxacin) clustered together (Fig. 2B).

### GuaB inhibition induces global transcriptional changes in *A. baumannii* and *E. coli*

The observation that GuaB inhibition resulted in different time-kill kinetics and distinct cell morphological changes in *A. baumannii* and *E. coli* prompted us to investigate whether there are differential regulatory responses induced by G6. We treated Ab19606 and MG1655 Δ*tolC* with 4× MIC of G6 for 1 h before RNA extraction and RNA-Seq. We chose this condition based on the fact that GuaB inhibition at 4× MIC mimicked deletion of *guaB* over the time tested (Fig. 1B through F), yet the bacterial cells remained intact and actively grew in size at this time point (Fig. 2A). As a control, we included ciprofloxacin that impedes DNA replication by inhibiting DNA topoisomerase and DNA gyrase. Treatment with G6 led to global transcriptome changes in both species, and the pattern of changes is distinct from that of ciprofloxacin (Fig. S3). The expression of 523 and 2108 genes were significantly changed (fold change >2, *P* < 0.05) after treatment of G6, representing 14.3% and 49.2% of total protein-coding genes in Ab19606 and in MG1655 Δ*tolC*, respectively (Table S2). We further performed KEGG pathway enrichment analysis for each strain and identified different sets of pathways that were significantly affected by GuaB inhibition in Ab19606 and in MG1655 Δ*tolC* (Table S3).

**Fig 3:**
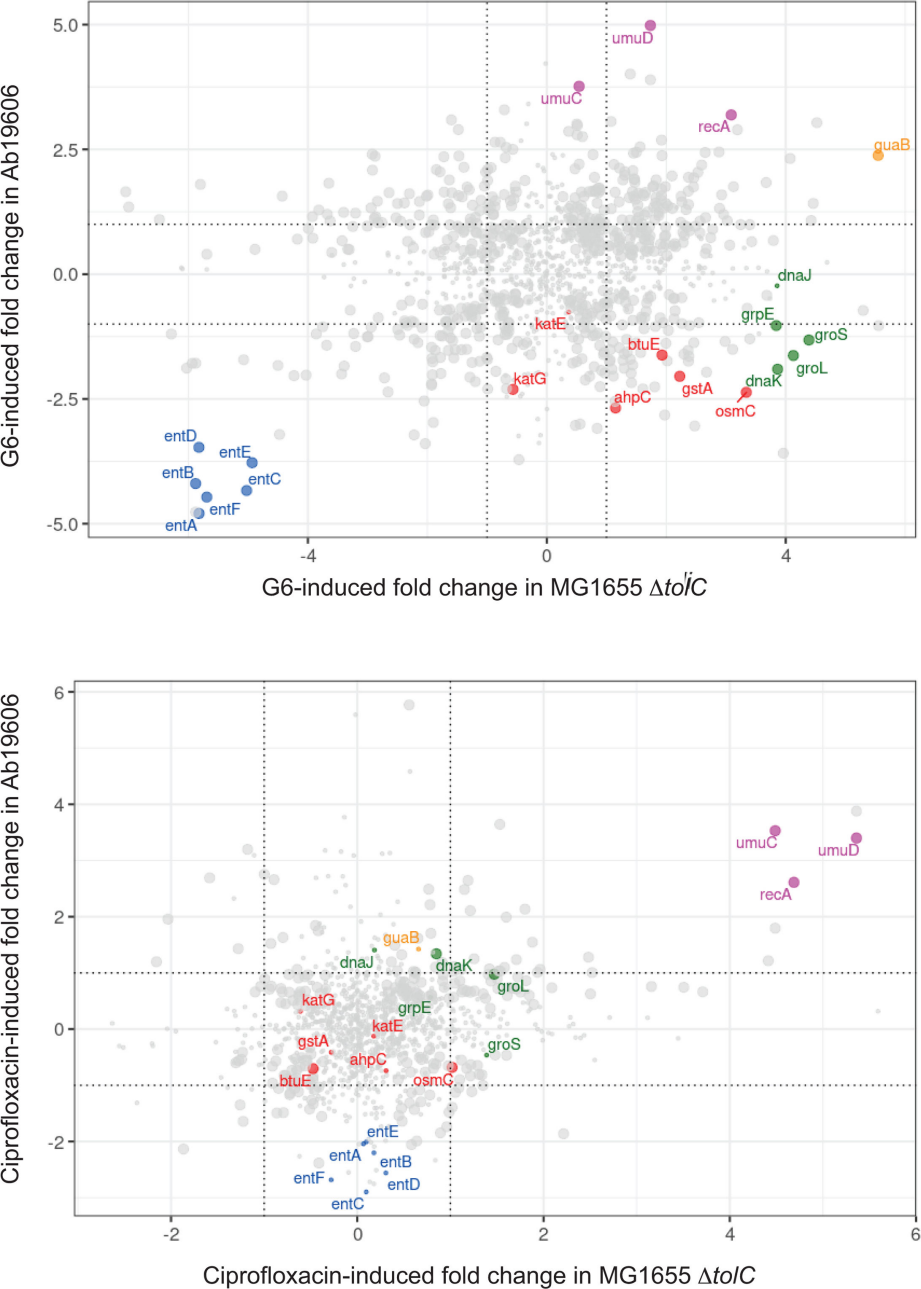
Transcriptional changes of orthologous genes in *A. baumannii* and *E. coli* induced by GuaB inhibition. Bacterial cells in log phase were treated with G6 or ciprofloxacin at 4× MIC for 1 h before RNA extraction. The log2 fold change in treated cells compared with untreated cells in *A. baumannii* (y-axis) and *E. coli* (x-axis). Each dot represents an ortholog. Large dots represent orthologs that have significant changes (*P* < 0.05) in both species; small dots represent orthologs that have significant changes only in one species or neither. The orthologs with similar functions are highlighted in color: the target *guaB* (orange); siderophore biosynthesis *ent* genes (blue); molecular chaperone genes (green); ROS detoxifying genes (red); DNA repair genes (purple).

To directly compare the effects of GuaB inhibition on *A. baumannii* and *E. coli* gene expression, we focused on the genes conserved in both species. To this end, we identified 1327 orthologs using a phylogenetic orthology inference-based OrthoFinder (37, 38). Among them, 502 and 714 orthologs have significant changes (fold change >2, *P* < 0.05) in Ab19606 and in MG1655 Δ*tolC*, respectively (Fig. 3; Table S4). For the rest of the study, we focused on the orthologs relevant to GuaB inhibition and bacterial viability.

### Distinct regulation of *de novo* purine biosynthesis in *A. baumannii* and *E. coli* upon GuaB inhibition

**Fig 4:**
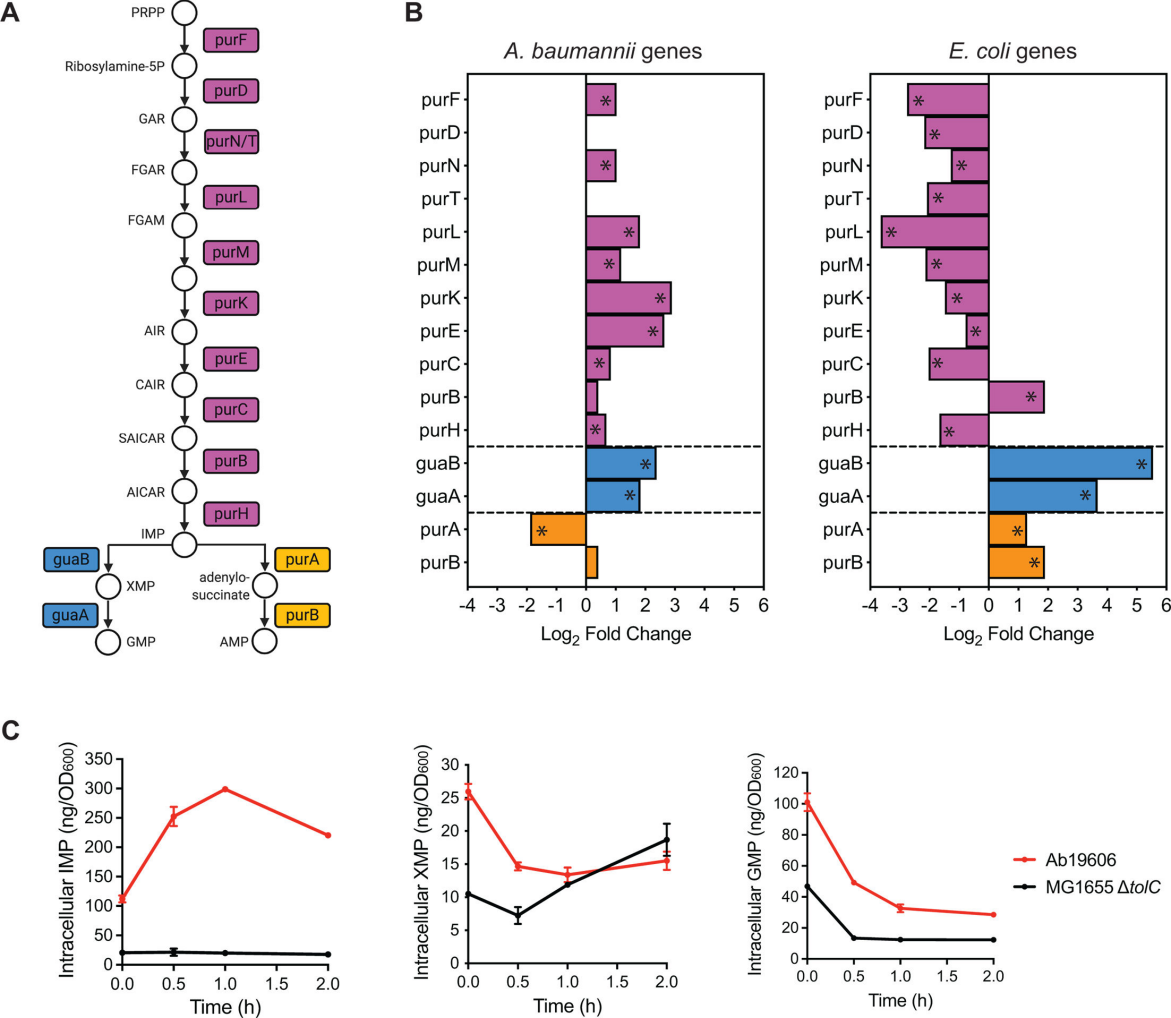
Distinct regulation of *de novo* purine biosynthesis in *A. baumannii* and *E. coli* upon GuaB inhibition. (**A**) Genes required for *de novo* purine biosynthesis are conserved in *A. baumannii* and *E. coli*. (**B**) Transcriptional changes of the genes in the *de novo* purine biosynthesis pathway were detected by RNA-Seq after GuaB inhibition. RNA-Seq data with significance (*P* < 0.05) were marked with *. (**C**) Bacterial intracellular IMP, XMP, and GMP changes after GuaB inhibition. Bacterial cells in log phase were treated with G6 at the concentration of 4× MIC before intracellular purines were extracted and measured by LCMS. The values were normalized against OD_600_, and the average and standard deviation of three independent experiments are shown.

We first examined changes in the expression of each conserved genes in the *de novo* purine biosynthesis pathway (Fig. 4A) and the intersecting metabolism pathways (Fig. S4). Intriguingly, with the exception of the *guaB* and *guaA* genes, which were upregulated in response to G6 in both species, the majority of genes in the pathway was regulated in an opposite manner between species. A general pattern emerged that upon exposure to G6, genes participating in steps before IMP formation were significantly upregulated in Ab19606 but downregulated in MG1655 Δ*tolC* (Fig. 4B). Moreover, genes whose products are involved in AMP biosynthesis, *purA* and *purB*, were upregulated in MG1655 Δ*tolC*, whereas in Ab19606 *purA* was downregulated, and *purB* was unchanged (Fig. 4B).

We expected that blocking biosynthesis at GuaB should lead to specific changes in metabolite levels, e.g., increased IMP (the substrate) and decreased XMP (the product). However, the gene regulation described above predicts that Ab19606 and MG1655 Δ*tolC* might have different changes. Indeed, intracellular IMP increased 2.7-fold in Ab19606 but remained unchanged in MG1655 Δ*tolC* (Fig. 4C). The level of XMP decreased in Ab19606 and increased in MG1655 Δ*tolC*. Ultimately, GuaB inhibition resulted in similar decreases in GMP levels in both species (Fig. 4C). Despite the conservation of the *de novo* purine biosynthesis pathway, it appears that the regulation of this pathway in response to GuaB inhibition is distinct between *A. baumannii* and *E. coli*.

### Siderophore biosynthesis and transport are downregulated following GuaB inhibition

**Fig 5:**
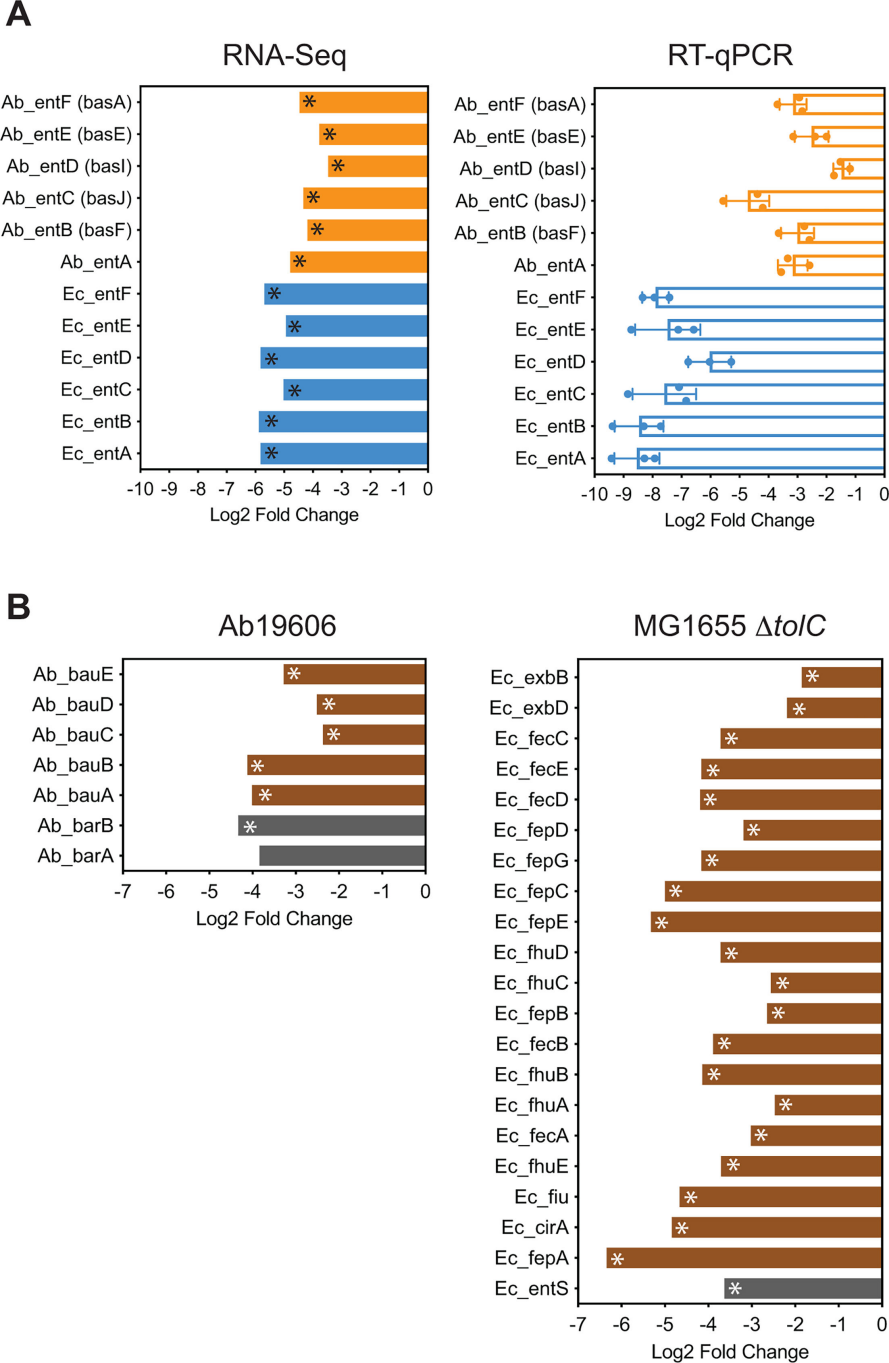
Siderophore biosynthesis and transport are greatly downregulated by GuaB inhibition. (**A**) RT-qPCR validated the downregulation of the genes required for DHBA biosynthesis. The averages and standard deviations of three independent experiments are shown. (**B**) Genes required for siderophore transport were downregulated. RNA-Seq data with significance (*P* < 0.05) were marked with *.

One of the most drastic transcriptional changes in response to GuaB inhibition is that genes required for siderophore biosynthesis were greatly downregulated in both Ab19606 and MG1655 Δ*tolC*. Siderophores are high-affinity iron-chelating compounds secreted by pathogenic bacteria for iron acquisition from host tissues. Iron is an essential cofactor for many cellular functions in bacteria and is one of the major limiting factors of pathogenic bacteria in the human host (39). *E. coli* produces enterobactin, a cyclic trimer of 2,3-dihydroxybenzoyl-L-serine (40). *A. baumannii* synthesizes up to 10 forms of siderophores, of which acinetobactin, a non-cyclic derivative of 2,3-dihydroxybenzoic acid (DHBA), is ubiquitous among different clinical isolates (41–45). Additionally, acinetobactin biosynthesis is essential for virulence of the *A. baumannii* strain Ab19606 (43). Although *E. coli* and *A. baumannii* synthesize different siderophores, both are derived from a common precursor, 2,3-dihydroxybenzoic acid (DHBA), and the genes required for DHBA biosynthesis are conserved in both species (*ent* genes for *E. coli* and *bas* genes for *A. baumannii*) (45, 46). Upon GuaB inhibition, expression of the *ent/bas* genes required for synthesis of siderophores decreased 4- to 32-fold. In general, the *E. coli* genes exhibited greater decreases than the *A. baumannii* genes upon GuaB inhibition (Fig. 5A). In comparison, after treatment with ciprofloxacin, expression of the *ent/bas* genes decreased similarly in Ab19606 but did not significantly change in MG1655 Δ*tolC* (Fig. 3; Table S3).

Following biosynthesis, acinetobactin is secreted via a siderophore efflux system consisting of the BarA and BarB proteins, and finally ferric–acinetobactin complexes enter bacterial cells via a transport system encoded by *bauA*, *bauB*, *bauC*, *bauD,* and *bauE* (44, 45). In *E. coli*, enterobactin is secreted by EntS (47) and taken up via a process composed of the energy-transducing TonB–ExbB–ExbD complex, specific outer membrane receptors (*fhuE, fhuA, fecA, fepA, cirA,* and *fiu*), periplasmic binding proteins (*fhuB, fecB,* and *fepB*), and ATP-binding cassette (ABC) transporters (*fhuCD, fecCDE,* and *fepCDEG*) in the inner membrane (39). Similar to the genes required for siderophore biosynthesis, we observed significant reductions in the expression of the siderophore efflux and transport systems in both Ab19606 and MG1655 Δ*tolC* (Fig. 5B).

### Effects of GuaB inhibition on stress responses

**Fig 6:**
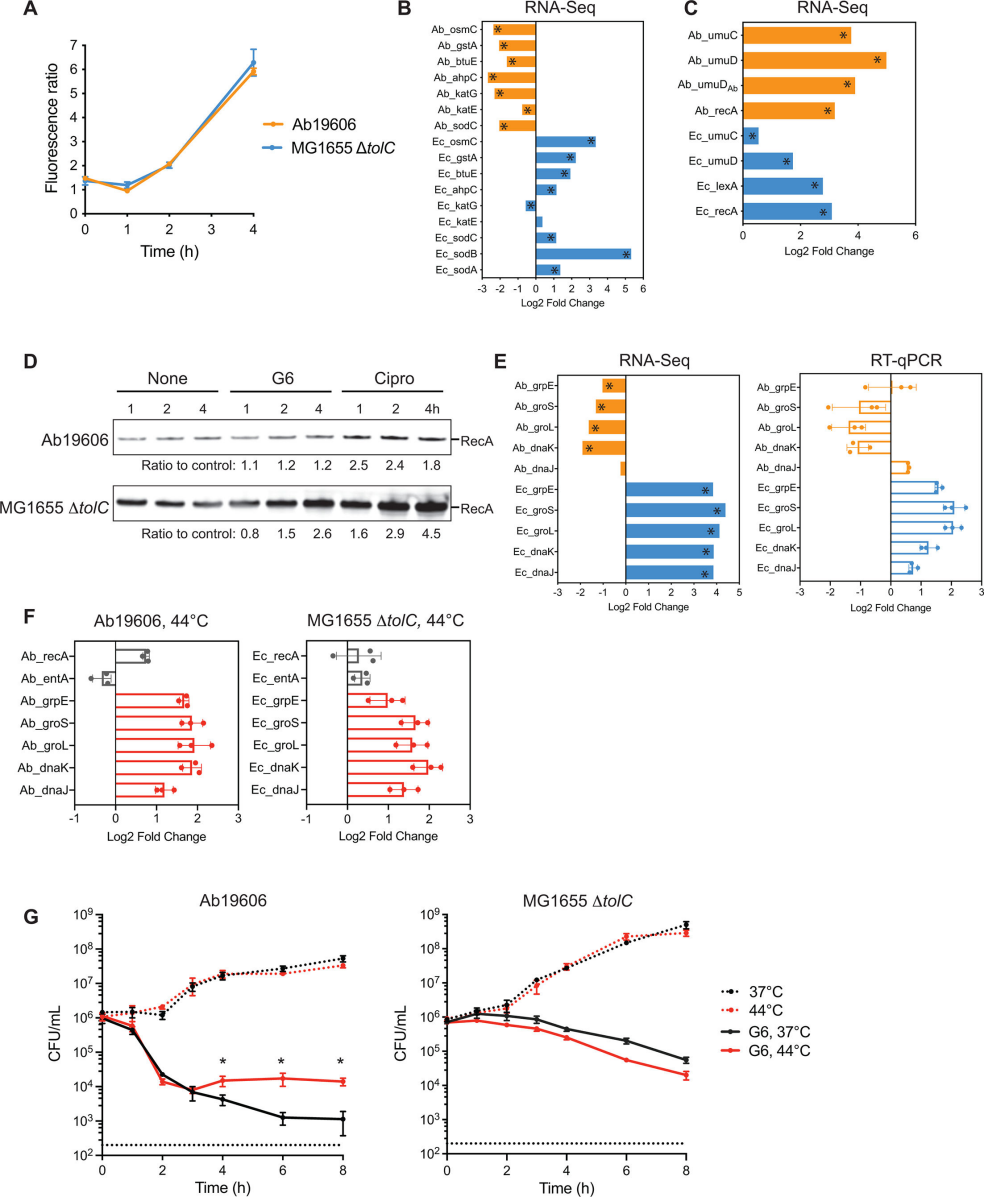
Effects of GuaB inhibition on stress responses. (**A**) GuaB inhibition activates production of ROS. Bacterial cells in log phase were treated with G6 at 4× MIC. ROS levels were measured by adding H_2_DCFDA 30 min before each time point. Fluorescence intensity was normalized against OD_600_. Fluorescence ratios of the treated and untreated cells are shown. Data are from three independent experiments. (**B**) *A. baumannii* and *E. coli* respond to oxidative stress differently. RNA-Seq detected transcriptional changes in genes encoding ROS detoxifying enzymes. (**C**) GuaB inhibition upregulates transcription of genes in the DNA repair pathway. (**D**) RecA levels following treatment with G6 or ciprofloxacin. Bacterial cells were treated with G6 or ciprofloxacin at 4× MIC for 1 h. The lysates were resolved on a 4%–12% SDS-PAGE gel. Loading volumes were normalized for equal total protein. RecA was detected by Western blotting using an antibody against *E. coli* RecA. The intensities of each band are quantified, and the ratios to their corresponding untreated controls are shown. (**E**) Transcription of molecular chaperone genes is upregulated in *E. coli* but downregulated in *A. baumannii* after GuaB inhibition. RNA-Seq data of five major chaperone genes were validated with RT-qPCR. The averages and standard deviations of three independent experiments are shown. (**F**) A higher temperature (44°C) induces upregulation of molecular chaperone genes. Ab19606 and MG1655 Δ*tolC* were grown to log phase at 37°C and shifted to 44°C for 30 min before RNA levels were measured by RT-qPCR. The averages and standard deviations of three independent experiments are shown. (**G**) Heat shock protects *A. baumannii* from GuaB inhibition. Ab19606 and MG1655 Δ*tolC* were grown to log phase at 37°C and shifted to 44°C for 30 min before addition of G6 at a concentration of 4× MIC. Viable cells were serially diluted and plated on M9 agar plate after exposure to G6. *, *P* < 0.05. RNA-Seq data with significance (*P* < 0.05) were marked with *.

Antimicrobial compounds and other lethal attacks stimulate the production of reactive oxygen species (ROS), which in turn contribute to bacterial cell death (48–56). To understand whether the GuaB inhibitor induces production of hydroxyl radicals, we directly measured hydroxyl radicals using a ROS-sensitive fluorescent dye. ROS levels showed no change at 1 h but increased by approximately sixfold at 4 h in both species (Fig. 6A). Interestingly, genes that encode enzymes involved in detoxifying ROS (57) changed in a different manner. In MG1655 Δ*tolC*, with the exception that the *katE* and *katG* catalase genes, which did not change or were slightly decreased, genes encoding superoxide dismutases (*sodA, sodB,* and *sodC*), alkyl hydroperoxide reductase (*ahpC*), and peroxidases (*btuE, gstA,* and *osmC*) were upregulated. The increased expression levels of these enzymes likely inactivate ROS and mitigate damage in MG1655 *ΔtolC*. By contrast, the Ab19606 orthologs of these genes were all downregulated (Fig. 6B).

*E. coli* mutant lacking the GuaB enzyme showed increased incorporation of xanthine and hypoxanthine into DNA and RNA (58), which we postulate could result in the activation of the DNA repair pathway to maintain genome integrity. Moreover, the increased levels of ROS can damage DNA. In *E. coli*, the DNA recombination/repair protein RecA is activated by single-stranded DNA and induces the auto-proteolytic activity of the repressor protein LexA, leading to de-repression of the SOS genes (59). The Ab19606 genome encodes a RecA protein, but lacks a homolog to LexA. UmuD_Ab_ was proposed as a functional homolog of LexA with major differences in the mechanism of regulating downstream genes (60–62). Our RNA-Seq data detected increased *recA* expression in Ab19606 and MG1655 Δ*tolC* (Fig. 6C). The *lexA* gene in MG1655 Δ*tolC* and *umuD_Ab_* in Ab19606 was also upregulated, as were the downstream genes *umuD* and *umuC* (Fig. 6C). However, Western blot analysis revealed that RecA protein levels had no apparent change over 4 h after addition of G6 in Ab19606, but had a > twofold increase in MG1655 Δ*tolC* (Fig. 6D).

Bacteria upregulate molecular chaperones, such as heat shock proteins, in response to environmental stresses, including antibiotics (48, 63, 64). Although the major molecular chaperones were dramatically upregulated (~16-fold) in MG1655 Δ*tolC* in the presence of G6, expression of the orthologs was decreased by as much as fourfold in Ab19606 (Fig. 6E). We postulate that this difference might contribute to the different time-kill kinetics of G6 in Ab19606 and MG1655 Δ*tolC* described earlier. To test this hypothesis, we performed a time-kill experiment with G6 under conditions that induced the major heat shock genes 2- to 4-fold (Fig. 6F). In Ab19606, the time-kill kinetics were unchanged in the first 3 h, but by 4 h after GuaB inhibition while the number of non-shocked bacteria continued to decline, the number of viable bacteria subjected to the heat shock remained unchanged. Notably, this protective heat shock effect was not observed in MG1655 Δ*tolC* (Fig. 6G). By comparison, all five heat shock genes were either upregulated or had no significant changes in both species in response to ciprofloxacin (Fig. S5A). Consistent with this observation, upregulating heat shock genes rendered neither species resistant to ciprofloxacin (Fig. S5B). This result suggests that upregulation of heat shock response could be a cellular response to ameliorate the lethal effects of GuaB inhibition in *A. baumannii*.

## DISCUSSION

We developed a series of IMPDH inhibitors that have up to 10^6^-fold selectivity for bacterial enzymes over the homologous human enzyme (Table 1), making them potential antibiotic candidates. Here, we showed that these antibacterial inhibitors induce cellular phenotypes that are distinct from other antibiotics (Fig. 2), consistent with their novel mechanism of action. Additional characterization of one of these inhibitors, G6, demonstrated that it is bactericidal against both wild-type *A. baumannii* and an *E. coli* efflux mutant (Fig. 1E and F). Although we cannot completely rule out that G6 could have other effects beyond inactivating GuaB, we have provided three lines of evidence in support of G6 as a specific inhibitor of GuaB in the *A. baumannii* Ab19606 and *E. coli* MG1655 Δ*tolC* cells. First, guanine supplementation rescued the *guaB* deletion mutants and the corresponding parental strains treated with bactericidal concentrations of G6 similarly in the time-kill assays. Specifically, guanine rescued the growth of *guaB* null mutants of Ab19606 (Fig. 1B) and MG1655 Δ*tolC* (Fig. 1D), as well as viability of both Ab19606 (Fig. 1E) and MG1655 Δ*tolC* (Fig. 1F) treated with G6. Second, the death kinetics of the *guaB* mutants and their corresponding wild type treated with G6 are largely similar. For Ab19606, the CFUs were reduced by 3-log reduction after 6 h for the *guaB* mutant and GuaB inhibition (compare Fig. 1B and E). For MG1655 Δ*tolC*, the *guaB* mutant and GuaB inhibition showed less than 1-log reduction after 6 h (compare Fig. 1D and F). These patterns hold true despite the difference in death kinetics between *A. baumannii* and *E. coli* further supports the specificity G6 as a specific *guaB* inhibitor. Third, the co-crystal structure of G6 with *A. baumannii* GuaB confirms that G6 binds to the GuaB enzyme directly (Fig. S1). Taken together, these results suggest that G6 is likely mediating the differential effects on *E. coli* and *A. baumannii* via direct on-target GuaB engagement.

To gain molecular insights into the physiological impact of GuaB inhibition in bacteria, we performed RNA-Seq analysis of the two strains treated with G6. Overall, we found that GuaB inhibition had an impact on a subset of protein-coding genes in Ab19606 (14.3%), whereas it affected nearly half of the MG1655 Δ*tolC* genome (49.2%). In contrast, ciprofloxacin had more comparable changes, 9.7% and 15.9% of total protein-coding genes had significant changes (fold change >2, *P* < 0.05) in Ab19606 and MG1655 Δ*tolC*, respectively (Table S2).

We identified pathways whose expression is altered in the same or opposite direction between the two species following GuaB inhibition. Genes involved in the highly conserved *de novo* purine biosynthesis pathway show strikingly different expressions between the two species (Fig. 4A and B). The difference in gene expression is associated with the accumulation of IMP in *A. baumannii* and XMP in *E. coli* over time (Fig. 4C). In *E. coli*, a global transcription factor PurR (purine repressor) regulates both purine and pyrimidine biosynthesis, including the *guaB* gene (65, 66). However, we did not identify a PurR ortholog in *A. baumannii* (Table S4). Taken together, this strongly suggests that *A. baumannii* regulates purine biosynthesis *via* a different mechanism. It is unknown whether an excess amount of intracellular IMP or XMP is detrimental to bacteria. The GuaB inhibitors used in this study are uncompetitive inhibitors that bind to the enzyme–substrate complex (GuaB–IMP complex) (5, 28). We postulate that the accumulated intracellular IMP in *A. baumannii* might provide an advantage in inhibiting GuaB.

In addition to disrupting purine biosynthesis, GuaB inhibition selectively affects amino acid biosynthesis. Genes for biosynthesis of tryptophan (*trpA*, *trpB*, *trpC*, and *trpD*), glutamate (*gltB *and *gltD*), and histidine (*hisA*, *hisB*, *hisC*, *hisD*, *hisF*, *hisG*, *hisH*, and *hisI*) were greatly downregulated in *E. coli*. In contrast, the orthologs in *A. baumannii* except *trpA* were increased or unchanged (Fig. S6). These contrasts were not evident in ciprofloxacin-treated bacteria (Fig. S6), suggesting that disruption of amino acid biosynthesis is unique to GuaB inhibition. How GuaB inhibition preferentially affects biosynthesis of these amino acids remains to be explored.

One similarity between *A. baumannii* and *E. coli* is the downregulation of the siderophore biosynthesis pathway and siderophore transport genes upon GuaB inhibition (Fig. 5). Iron is essential for bacterial growth and plays a key role in virulence in the human host. This downregulation likely leads to iron starvation and consequently contributes to bacterial cell death. A major difficulty in developing novel antibiotics against Gram-negative pathogens is the outer membrane, which is a permeability barrier to many antimicrobials. The siderophore-dependent iron transport pathway has been used as a Trojan horse strategy in which antibiotics are covalently linked to siderophores and actively transported into bacterial cells. This strategy can be designed to target defined bacteria as specific siderophores and transport systems are exploited (67). However, in light of our observation, this strategy might not be feasible for antimicrobials that dramatically reduce siderophore expression.

Bacteria have evolved a variety of stress responses to mitigate the effects of environmental insults, including antibacterial compounds. Here, we examined three stress responses: responses to ROS, the DNA repair pathway, and molecular chaperones which prevent proteins from misfolding, aggregation and degradation. Although *A. baumannii* and *E. coli* display similar increases in ROS levels in response to the GuaB inhibition, surprisingly, the genes involved in detoxifying ROS are regulated in opposite directions (Fig. 6A and B). Secondly, we found that the genes in the DNA repair pathway are elevated in both species. However, an increased RecA protein level was observed only in *E. coli* (Fig. 6C and D). Finally, we observed completely distinct gene expression changes for molecular chaperones. Five major heat shock genes increased expression in *E. coli* but decreased in *A. baumannii* (Fig. 6E). Interestingly, when we used a higher temperature to induce expression of heat shock genes, *A. baumannii* became partially resistant to the GuaB inhibitor (Fig. 6F and G). Collectively, our findings suggest that *E. coli* employs stress responses more readily to ameliorate the impact of GuaB inhibition and therefore survives for a longer time compared with *A. baumannii*. Lack of proper stress responses to GuaB inhibition in *A. baumannii* highlights that the GuaB inhibitor G6 has particular advantages as an antimicrobial against this pathogen.

## MATERIALS AND METHODS

### Bacterial strains, plasmids, and growth conditions

Bacterial strains used in this study are listed in Table S5. All *A. baumannii* and *E. coli* strains were grown in minimal media M9 (1× M9 salts, 0.5% casamino acids, 0.1 µg/mL thiamine hydrochloride, 1 mM MgSO_4_, 0.1 mM CaCl_2_, 0.2% glucose) except the *guaB* null strains, which were grown in M9 media supplemented with 100 µM guanine. *S. aureus* wild-type was grown in RPMI 1640 or on cation-adjusted Mueller–Hinton agar. *S. aureus guaB* null was grown in RPMI 1640 or on cation-adjusted Mueller–Hinton agar supplemented with 100 µM guanine. All strains were grown at 37°C unless stated otherwise.

The plasmid pBla-*sfGFP* was from a previous study (68). pWH1266 containing an *A. baumannii* ori sequence was obtained from ATCC. The *sfGFP* sequence in pBla-*sfGFP* was subcloned in pWH1266 at the BamHI site to generate pWH1266-*sfGFP*. pBla-*sfGFP* and pWH1266-*sfGFP* were transformed to MG1655 Δ*tolC* and Ab19606, respectively. The GFP-expressing strains were grown in the presence of 50 µg/mL carbenicillin to maintain the plasmids.

### Synthesis of GuaB inhibitors

Detailed methods are provided in the Supplemental Material.

### IC_50_ measurement

For the *E. coli* GuaB enzyme, GuaB inhibitors (dissolved in DMSO) or DMSO were dispensed as serial dilutions on a Labcyte Echo555 acoustic dispenser (Beckman Coulter) to a 384-well clear bottom plate (μClear, Grenier 781090) immediately before use. The total volume of DMSO was either 200 or 400 nL per well, with a final DMSO concentration of either 0.5% or 1%. Recombinant *E. coli* GuaB (GuaB*^Ec^*, 1 nM), substrate IMP (70 µM, Sigma I4625), and NAD + cofactor (700 µM, Sigma N1636) were diluted in Assay Buffer (50 mM Tris, pH 8.0, 100 mM KCl, 3 mM EDTA, 0.002% Tween-20, 1 mM DTT), and subsequently added to the assay plate. GuaB-mediated NADH production was monitored by measuring absorbance kinetically at 1-min intervals at 340 nm.

For *A. baumannii* and *S. aureus* GuaB enzymes, GuaB inhibitors or DMSO were dispensed to a 384-well solid white plate (Proxiplate, Revvity, 6008280) as described above. The total volume of DMSO was 100 nL per well, with a final DMSO concentration of 1%. Recombinant GuaB (0.2 nM and 0.6 nM for GuaB*^Ab^* and GuaB*^Sa^*, respectively), substrate IMP (100 and 60 µM for GuaB*^Ab^* and GuaB*^Sa^*, respectively) and NAD + cofactor (1,000 and 340 µM for GuaB*^Ab^* and GuaB*^Sa^*, respectively) were diluted in Assay Buffer and subsequently added to the assay plate. GuaB-mediated NADH production is detected with the addition of NAD(P)H-Glo detection system (Promega, G9062). In the presence of NADH, a reductase/luciferase enzyme cascade produces luminescence proportional to the amount of NADH.

Absorbance or luminescence values were normalized to no inhibition and 100% inhibition controls, and the normalized data were fit to a four-parameter sigmoidal equation to determine IC_50_ values for each inhibitor (Table S1).

### Minimum inhibitory concentration (MIC) assays

Broth MICs of *A. baumannii* and *E. coli* were determined in M9 media, broth MICs of *S. aureus* were determined in RPMI 1640, according to a standard Clinical and Laboratory Standards Institute (CLSI) protocol.

### Time-kill curves

The *guaB* null strains and the corresponding wild-type strains were grown to exponential phase in broth media supplemented with 100 µM guanine (*A. baumannii* and *E. coli* strains in M9 media, *S. aureus* stains in RPMI 1640). The bacteria were spun down and immediately resuspended in fresh media with or without guanine to 10^6^ CFU/mL. The bacterial cultures were grown at 37°C. At each time point, dilutions of the bacterial cultures were plated on agar media (*A. baumannii* and *E. coli* strains on M9 agar, *S. aureus* stains on cation-adjusted Mueller–Hinton agar) with or without guanine. The plates were incubated at 37°C and colonies were counted after overnight growth to determine the number of viable bacteria.

For GuaB inhibition time-kill assays, the strains were grown and diluted to 10^6^ CFU/mL in their corresponding broth media with or without guanine. G6 (10 mM in DMSO) or equal volumes of DMSO was added to 2-mL aliquots of bacterial cultures, and the cultures were grown at 37°C. At each time point, 100 µL of the cultures was retrieved, and dilution of the cultures was plated on agar plates with or without guanine for CFU numeration as described above.

### Time-lapse microscopy

Ab19606 and MG1655 Δ*tolC* strains expressing cytoplasmic GFP were grown to exponential phase in M9 media containing 50 µg/mL carbenicillin at 37°C and treated with G6 at 4 x MIC (12 µM for Ab19606 and 16 µM for MG1655 Δ*tolC*). Bacterial cells were then immediately placed between a cover slip and a 1% agarose pad containing the same concentration of G6 for imaging. The cells were maintained at 37°C during imaging with a stage top chamber (Okolab Inc.) and imaged on a Nikon Eclipse Ti inverted confocal microscope (Nikon Instruments Inc.) coupled with a UltraVIEW VoX (PerkinElmer Inc.) and a 100× (NA 1.40) oil-immersion objective. Images were captured using an ORCA-Flash 4.0 CMOS camera (Hamamatsu Photonics), collected using Volocity software (Quorum Technologies) and processed using Fiji (69).

### Bacterial phenotypic profiling

Bacterial samples were prepared for high throughput imaging as described previously with modifications (36). The GuaB inhibitors and standard antibiotics were serially diluted in a 384-well assay plate using Echo Acoustic Liquid Handler (LabCyte). The total volume of DMSO is 75 nL each well (final concentration 0.5%). Ab19606 and MG1655 Δ*tolC* bacteria were grown to exponential phase in M9 media at 37°C and diluted to 0.4 OD_600_/mL with prewarmed M9 media. Subsequently, 30 µL of the bacterial suspensions was dispensed into each well and incubated for two doublings (1 h and 2 h for *E. coli* and *A. baumannii*, respectively) at 37°C. At 30 min before the end of the incubation, 10 µL of pre-warmed M9 media containing Sytox Green (Life Technologies) and FM4-64 (Invitrogen) dyes was added to a final concentration of 1 µM and 2.5 µg/mL, respectively. After two doublings at 37°C, Sytox Green and FM4-64 were removed by adding 40 µL PBS per well, centrifuging plate for 1 min at 1000 rpm, and subsequently aspirating the supernatant with 10-µL volume remaining per well. Subsequently, 10 µL 4% paraformaldehyde in PBS containing 4 µg/mL DAPI (Promo Kine) was added, and the samples incubated for 30 min in the dark at room temperature. The samples were then washed twice by adding 60-µL PBS, centrifuging and aspirating supernatant to the 10 µL remaining volume. The samples were resuspended by adding 40 µL PBS, and 18 µL was transferred to a 384-well black/clear amine coated imaging plate (Corning). All the above steps were carried out using a Multidrop Combi reagent dispenser (Thermo Fisher) and a Biomek FX robotic workstation (Beckman Coulter). The samples were imaged on an Opera Phenix high content screening system with a 63 x water objective (Perkin Elmer).

Images were analyzed using a workflow created with Harmony building blocks (Perkin Elmer) to extract features describing shape, intensity, and texture (SER Features) for each of the fluorescent channels. A total of 106 features were extracted and analyzed with a workflow created in KNIME with R script nodes as follows: SMAD-normalization relative to DMSO wells, outlier removal by interquartile range filter (1.5 × 5th and 95th percentiles), selection of the 20 most informative features using R package ‘mRMR’ applied to the reference antibiotic samples; Euclidean distance relative to DMSO. For each compound, the lowest effective dose (LOED) was determined as having a Euclidean distance of >3 sd from the mean of DMSO wells. Doses corresponding to LOED and the next two higher doses were selected and clustered using the R package ‘Rtsne’.

### GuaB inhibition and RNA isolation

*A. baumannii* strain Ab19606 and *E. coli* strain MG1655 Δ*tolC* were grown to log phase in M9 media and diluted with fresh M9 media to 0.1 OD_600_/ml. G6 or ciprofloxacin was added to 4× MIC (G6: 12 µM for Ab19606 and 16 µM for MG1655 Δ*tolC*; ciprofloxacin: 4 µg/mL for Ab19606 and 0.016 µg/mL for MG1655 Δ*tolC*). After incubation with shaking for 1 h at 37°C, the bacteria were immediately spun down and resuspended in the RNAprotect bacteria reagent (Qiagen). RNA was extracted using RNeasy spin columns (Qiagen) according to the manufacturer’s protocol.

### RNA-Seq

Quality control of RNA samples was performed to determine RNA quantity and quality before their processing by RNA-Seq. The concentration of RNA samples was determined using NanoDrop 8000 (Thermo Scientific), and the integrity of RNA was profiled on 2100 Bioanalyzer (Agilent Technologies). Ribosomal RNA depletion was performed using a Ribo-Zero rRNA Removal Kit (Bacteria) (Illumina, San Diego, CA) according to the manufacturer’s instructions. RNA-Seq library generation was continued using the Truseq RNA Library Prep Kit v2 (Illumina) starting at the “Elute, Prime, Fragment” step and was completed according to the manufacturer’s protocol. The size of the libraries was confirmed using 4200 TapeStation and High Sensitivity D1K screen tape (Agilent Technologies), and their concentration was determined by qPCR-based method using a library quantification kit (KAPA). The libraries were multiplexed and sequenced on Illumina HiSeq4000 (Illumina) to generate approximately 20 million of single end 50 base pair reads.

Ribosomal RNA contamination was removed from the fastq sequence files, and reads were filtered for quality, keeping only reads where at least 70% of the cycles had Q scores ≥ 23. Single-end 50bp reads were aligned to the *E. coli* K12 MG1655 and *A. baumannii* ATCC19606 genome using GSNAP version 2013–11-01 with the following parameters: -M 2n 10 -B 2 -i 1 --pairmax-dna=1000 --terminal-threshold=1000 --gmap-mode=none --clip-overlap. Uniquely mapped reads were used to obtain read counts and RPKMs per gene. Differential expression analysis was performed with voom+limma. Orthologs between strains were detected using OrthoFinder (37). To simplify our analysis, we limited each orthologous group to one gene in *E. coli* but allowed for the presence of paralogs (i.e., multiple orthologous genes) in *A. baumannii*. Raw and processed RNA-seq data for *A. baumannii* and *E. coli* have been deposited on the Gene Expression Omnibus platform with the accession number GSE275261 .

### RT-qPCR

cDNA was synthesized using the SuperScript III First-Strand Synthesis System (Invitrogen). qPCRs were performed using TaqMan Universal PCR Master Mix (Applied Biosystems) and gene-specific primers and probes (IDT, Table S6) on a 7500 Real Time PCR instrument (Applied Biosystems). Relative gene expression was analyzed using Applied Biosystems software and the 2^-ΔΔCT^ method (70, 71).

### Quantification of bacterial intracellular purines by LC-MS/MS

*A. baumannii* strain Ab19606 and *E. coli* strain MG1655 Δ*tolC* were grown to log phase in M9 media. G6 was added to 4× MIC. At each time point, bacterial density (OD_600_) was measured, and 15 mL of bacterial cultures was collected. To extract intracellular purines, bacterial pellets were suspended in 300 µL of 80:20 methanol:water (v/v), subsequently incubated in a dry ice methanol bath for 15 min, and centrifuged at 14,000 rpm for 5 min at 4°C. The extraction was repeated twice. In the third extraction, an additional 15 min of sonication in an ice bath was carried out before centrifugation. The cell extracts were pooled for the following analysis.

The analytical reference standards of guanosine 5′-monophosphate (GMP) and inosine 5′-monophosphate (IMP) were purchased from J&K Scientific (Beijing, China), xanthosine 5′-monophosphate (XMP) was purchased from USBiological Life Sciences (MA, USA), and the internal standard (IS), guanosine-^15^N_5_ 5′-monophosphate (GMP-15N5), was purchased from Sigma-Aldrich (MO, USA). The cell extract samples were prepared for analysis by mixing a 90-µL aliquot of cell lysate and 10 µL of 50:50 methanol:water (v/v) containing the internal standard mixture into a 96-well plate. The samples were vortex-mixed at room temperature for 1 min and ready for analysis. The concentrations of GMP, IMP, and XMP in *E. coli* and *A. baumannii* were determined by a high-performance liquid chromatography-tandem mass spectrometry (HPLC-MS/MS) assay. A Shimadzu Nexera UPLC system coupled to a QTRAP 5500 SCIEX in positive ion ESI mode (SCIEX, Foster City, CA) was used for sample analysis. The mobile phases were 20 mM ammonium acetate and 20 mM ammonium hydroxide in water (A) and 100% acetonitrile (B). A gradient method was used, and the total flow rate was 1.0 mL/min. The samples were injected into a Phenomenex Luna NH2 (50 × 2.0 mm, 3 µm) analytical column with a total run time of 4 min. The analysis commenced with the mobile phase set at 30% B. It was ramped linearly down to 0% B over a period of 2.5 min and held at 0% B until 3.3 min, then quickly adjusted back to 30% B, and maintained at 30% B for the rest of the time, totaling a 4-min run. Data were acquired using multiple reactions monitoring (MRM) in positive ion electrospray mode. A duplicate set of calibration curve was prepared in 50:50 methanol:water (v/v) with known concentrations of each analyte at 11 levels and included in each HPLC-MS/MS run of the cell extract samples. The lower limit of quantitation (LLOQ) is 7.81 ng/mL for GMP and IMP and 15.6 ng/mL for XMP. The peak area ratio of each analyte to IS (GMP-15N5) vs. concentration of the analyte was fitted with a weighted quadratic curve to determine the unknown analyte concentrations in the cell extract samples.

### ROS measurements

*A. baumannii* strain Ab19606 and *E. coli* strain MG1655 Δ*tolC* were grown to log phase and adjusted to 10^7^ CFU/mL in M9 media. The bacterial suspensions were treated with G6 at 4× MIC at 37°C. ROS levels were measured by adding 5 µM of 2′,7′‐dichlorodihydrofluorescein diacetate (H_2_DCFDA, Invitrogen) to the bacterial suspension 30 min before each time point. The fluorescence was determined using a SpectraMax M5 microplate reader (Molecular Devices) with excitation/emission wavelengths of 485/530 nm. The fluorescence intensity (FI)/OD_600_ was calculated to normalize the fluorescence with respect to growth. Untreated cells were processed similarly and used as the control.

### Western blot

Bacterial cells were lysed in a 1 × LDS sample buffer (Invitrogen) containing 4% 2-mercaptoethanol by boiling for 10 min. Aliquots of lysates containing equal amounts of total protein were resolved by SDS-PAGE on a 4%–12% Tris-Bis gel (Invitrogen). Proteins were transferred to nitrocellulose membranes using the iBlot two gel horizontal transfer system (Invitrogen). RecA was detected using a rabbit anti-RecA antibody (GeneTex) followed by a secondary antibody obtained from LI-COR. Images were acquired and quantified using the Odyssey CLx imaging system (LI-COR).

### GuaB purification, crystallization, and structure determination

The *A. baumannii* GuaB with CBS domain deletion protein (M1-G488_ΔM93-R201) was expressed, purified, and crystallized as described (28). In short, the purified *A. baumannii* GuaBΔCBS protein (21 mg/mL) was incubated with 5 mM IMP before mixing 1:1 with reservoir solution (0.1 M HEPES, pH 7.5, 20% isopropanol and 10% PEG4000). The complexes with small-molecule inhibitors were obtained by soaking the crystals 1–2 nights in artificial mother liquor with 5 mM of compound G6 and 5 mM IMP, then dipping into cryo-protectant solution consisted of 0.1 M HEPES pH 7.5, 10% PEG4000, 20% ethylene glycol, 2.5 mM IMP, and 2.5 mM compound, before flash freezing in liquid nitrogen. Data sets were collected at the ALS beamline 5.0.2. The structure was determined by molecular replacement using an in-house model, originally derived from published GuaB CBS-deletion structures (e.g. *V. cholera* PDB 4I × 2). The *A. baumannii* GuaB crystallized in space group P2_1_ with eight molecules per asu. The structure was manually rebuilt using COOT (72) and refined with Phenix (73) and Buster (74). Molecular graphics were prepared with PyMOL (Schrödinger, LLC, 2010). The refined structural model and structure factors (Table S7) are deposited with PDB code 9C4M.

## SUPPLEMENTAL MATERIAL

Supplemental material jb.00102-24-s0001.pdf Supplemental figures, figure legends, synthesis of GuaB inhibitors, and tables S1, S5, S6, and S7.

Table S2 jb.00102-24-s0002.xlsx Transcriptional changes in *A. baumannii* and *E. coli*.

Table S3 jb.00102-24-s0003.xlsx KEGG pathway enrichment analysis of transcriptional changes.

Table S4 jb.00102-24-s0004.xlsx Transcriptional changes of the orthologs in *A. baumannii* and *E. coli*.

ASM does not own the copyrights to Supplemental Material that may be linked to, or accessed through, an article. The authors have granted ASM a non-exclusive, world-wide license to publish the Supplemental Material files. Please contact the corresponding author directly for reuse.
