# Supplementary material for: Differential effects of inosine monophosphate dehydrogenase (IMPDH/GuaB) inhibition in Acinetobacter baumannii and Escherichia coli
Source: J Bacteriol. 2024 Sep 5;206(10):e00102-24. doi: 10.1128/jb.00102-24 (PMC11500612; doi:10.1128/jb.00102-24)
Supplement: Supplemental material — Supplemental figures, figure legends, synthesis of GuaB inhibitors, and tables S1, S5, S6, and S7. [file jb.00102-24-s0001.pdf]

Supplemental Material

Supplemental Figures.

Supplemental Figure Legends.

Synthesis of GuaB inhibitors.

Table S1. Additional information on IC_{50} measurement.

Table S2. Transcriptional changes in *A. baumannii* and *E. coli* after treatment of GuaB inhibitor G6 and ciprofloxacin (Excel file).

Table S3. KEGG pathway enrichment analysis of transcriptional changes in *A. baumannii* and *E. coli* after treatment of GuaB inhibitor G6 (Excel file).

Table S4. Transcriptional changes of the orthologs in *A. baumannii* and *E. coli* after treatment of GuaB inhibitor G6 and ciprofloxacin (Excel file).

Table S5. Bacterial strains used in this study.

Table S6. RT-qPCR primers used in this study.

Table S7. Structure data collection and refinement statistics.

Figure S1. The crystal strcture of *A. baumannii* GuaB bound to IMP and G6.

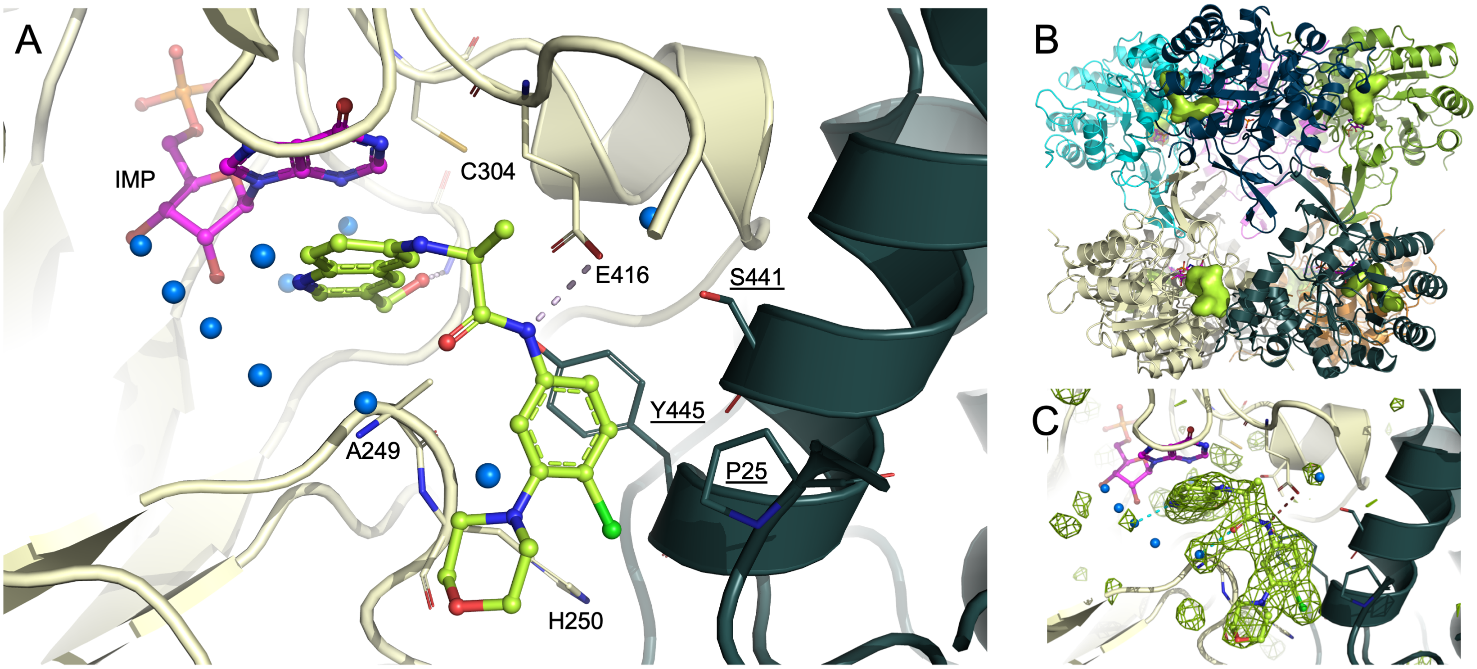

Figure S2. *S. aureus* time-kill kinetics upon GuaB inhibition.

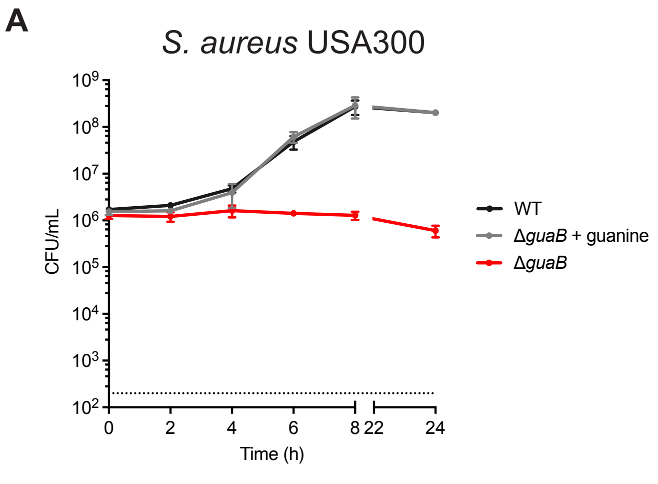

Figure S3. Hierarchical clustering of gene expression after the treatment of G6 or ciprofloxacin.

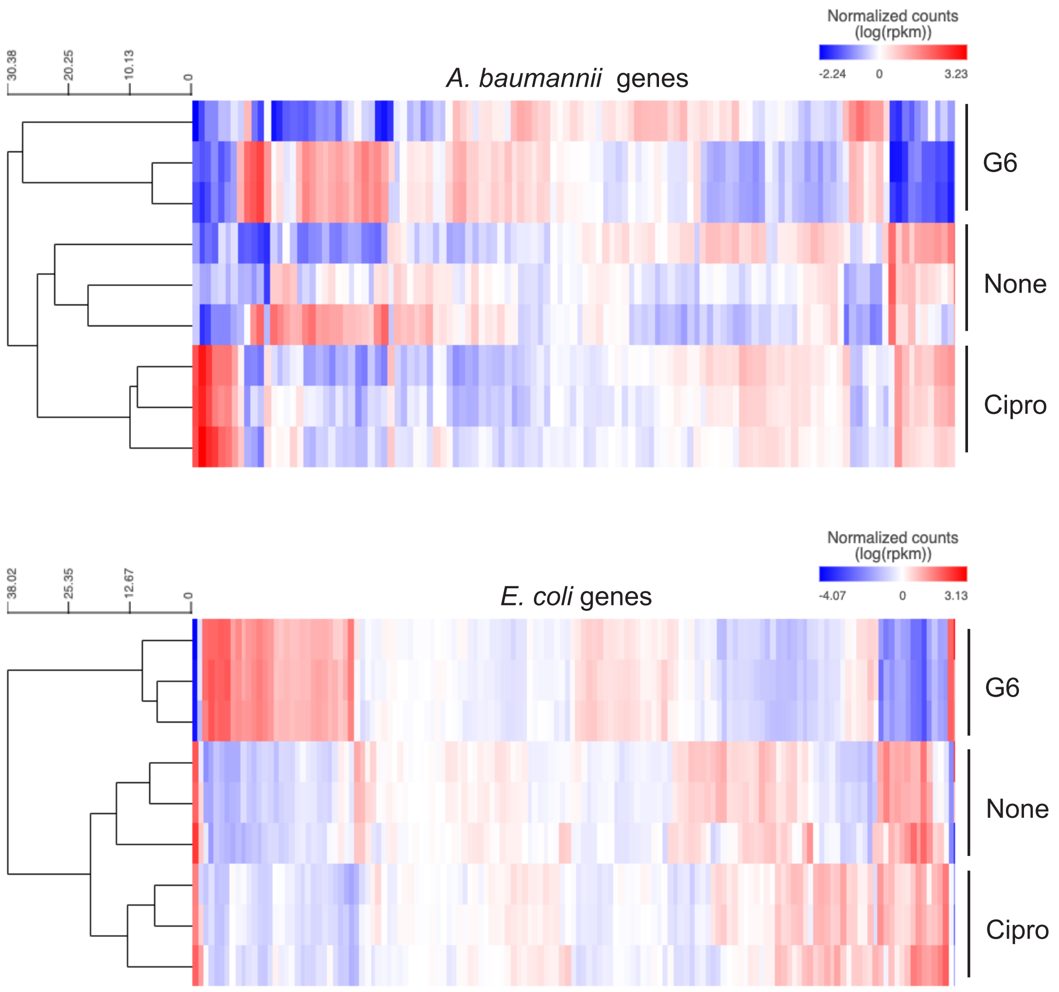

Figure S4. Changes in gene expression mapped to KEGG pathways.

A. baumannii (Ab19606) transcription changes after GuaB inhibition

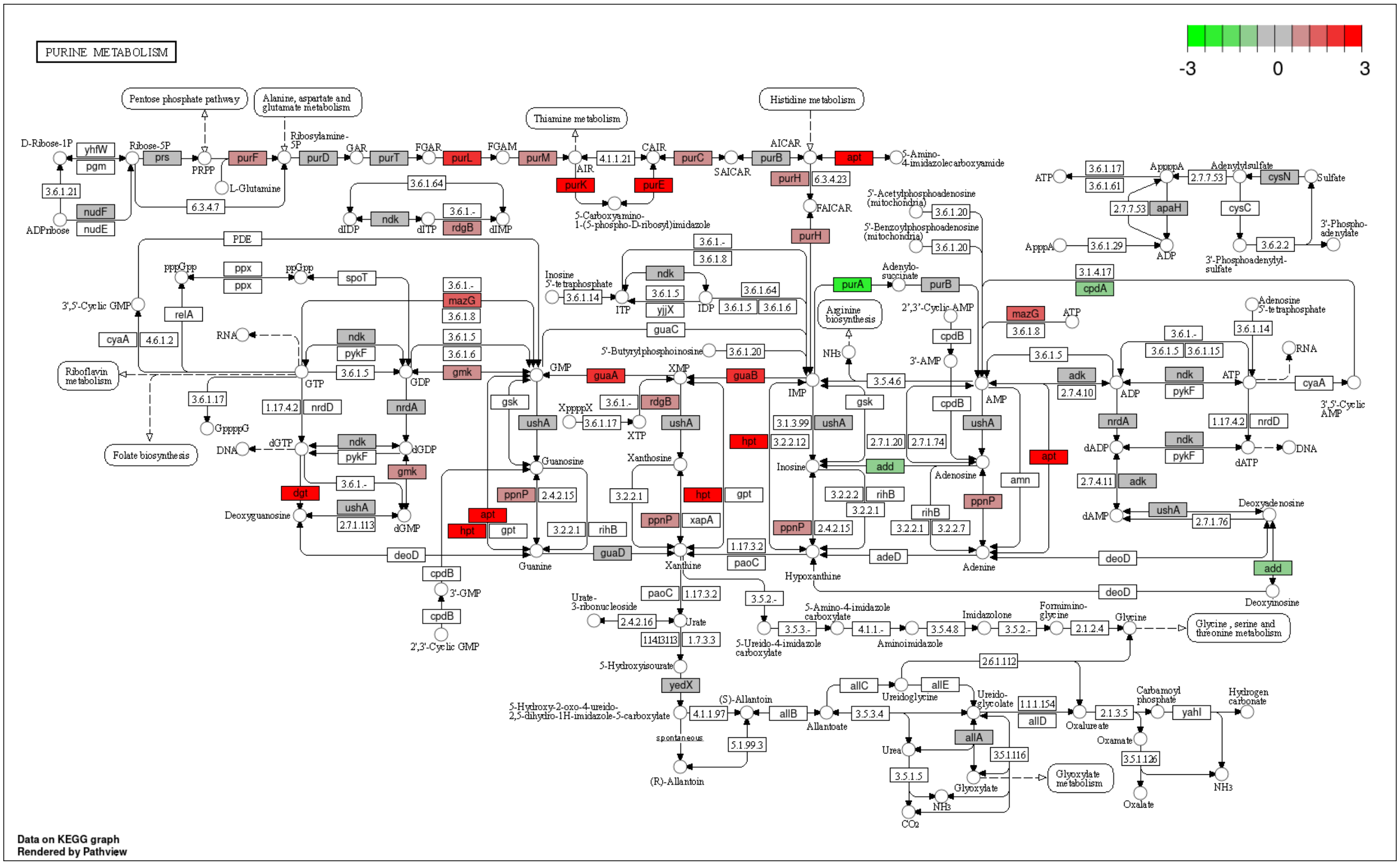

E. coli (MG1655 Δ*tolC*) transcription changes after GuaB inhibition

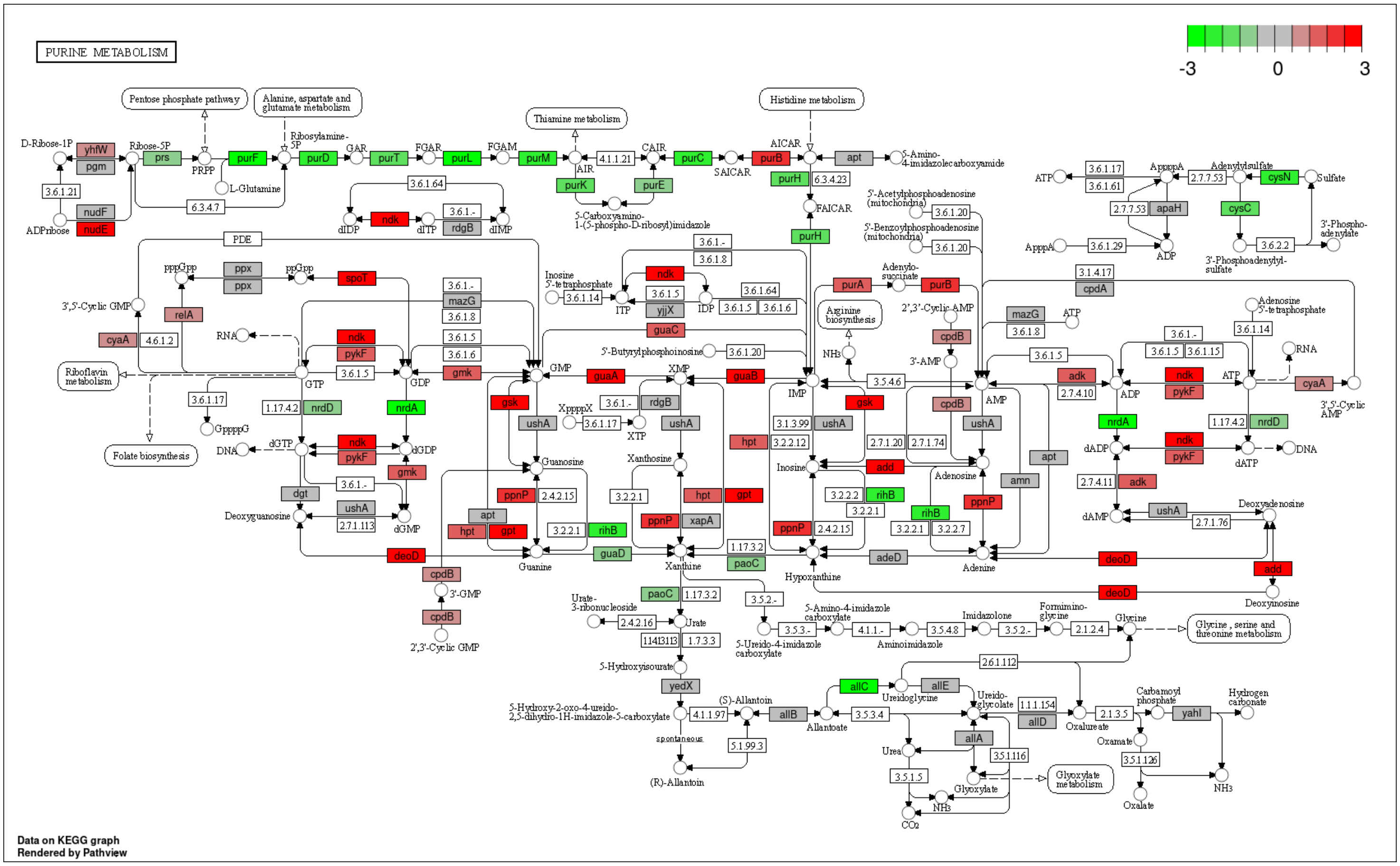

Figure S5. Upregulation of molecular chaperone genes does not result in resistance to ciprofloxacin in either species.

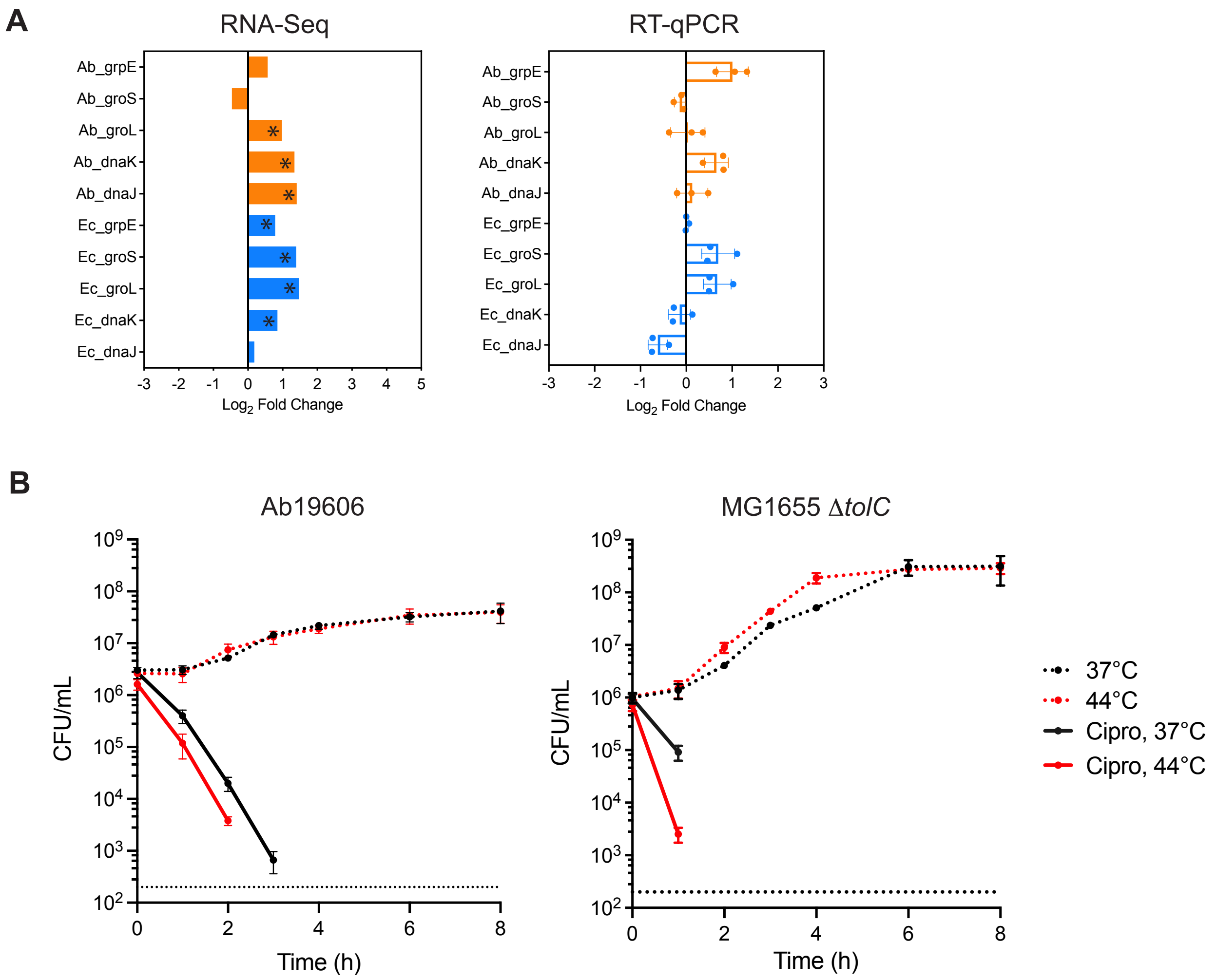

Figure S6. GuaB inhibition leads to opposite transcription changes in genes required for tryptophan, glutamate and histidine biosynthesis in *A. baumannii* and *E. coli*.

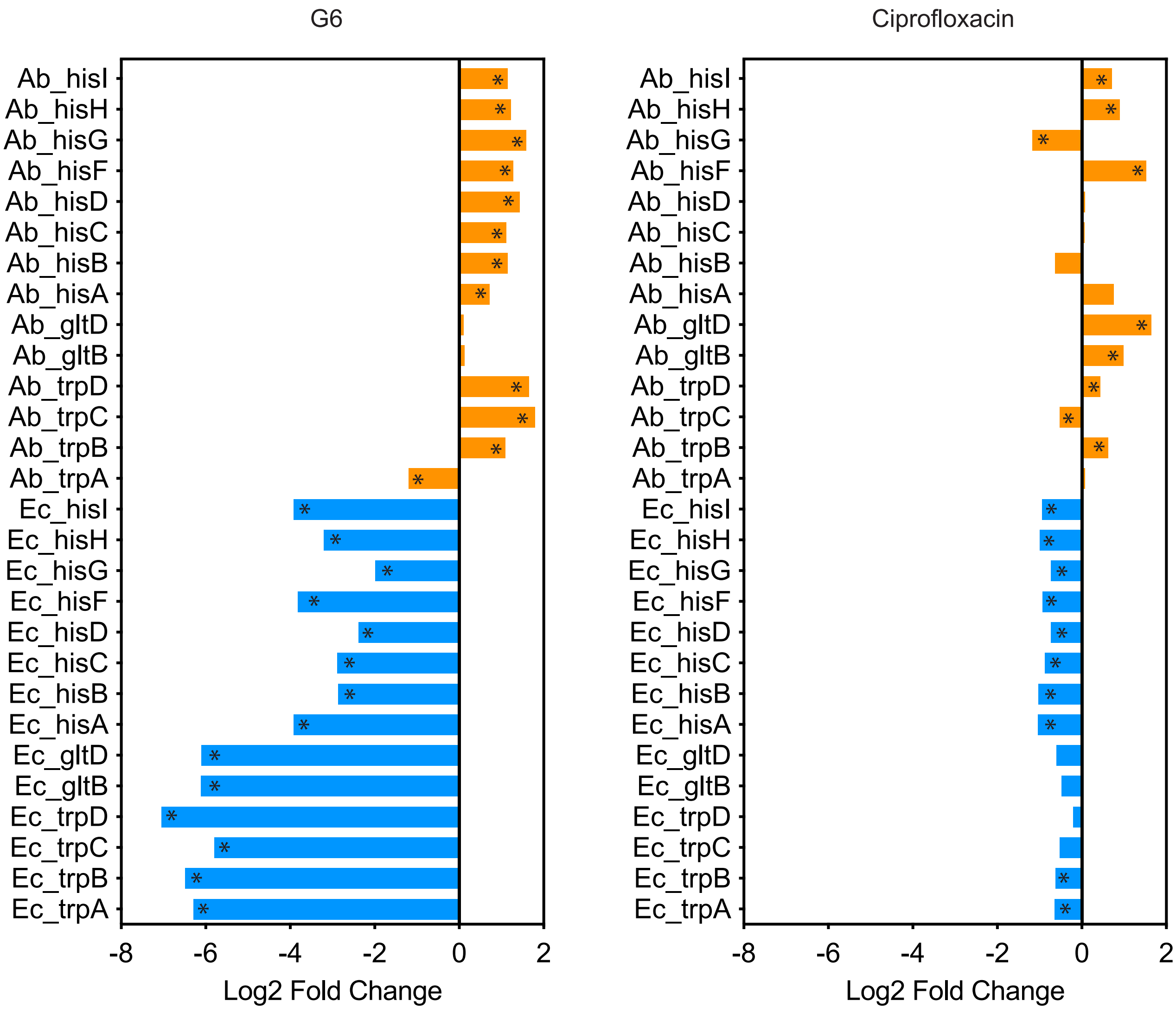

Supplemental Figure Legends

Figure S1. The crystal structure of *A. baumannii* GuaB bound to IMP and G6. (A) A detailed view of a representative binding pocket from the octamer of the crystallographic asymmetric unit showing key interactions of G6 (green sticks) with IMP (magenta sticks) and surrounding residues from two GuaB neighboring chains (chain A: ivory, chain B: dark teal, underlined labels). Proximal ordered water molecules are depicted as blue spheres, and hydrogen bonds shown as dashed lines. From this perspective the foreground would be occupied by the GuaB chain A "flap" feature, which is here dynamic and disordered as common to many IMPDH structures. (B) An overview of the octamer in the asymmetric unit of the crystal, shown as two tetramer layers inverted relative to each other. Ligand binding is observed between each pair of partners, with the G6 ligand here represented with molecular surface in bright green. (C) An Fo-Fc density representation prior to ligand inclusion in the model, here overlayed with the final refined structure, shows the quality of indication for the full envelope of the ligand.

Figure S2. *S. aureus* time-kill kinetics upon GuaB inhibition. (A) Time-kill assays of *S. aureus* USA300 Δ*guaB*. The *guaB* null and corresponding wild-type strains were grown to log phase at 37°C in RPMI 1640 supplemented with 100 μM guanine and shifted to RPMI 1640 with or without guanine. Viable cells were serially diluted and plated on Mueller-Hinton agar plates supplemented with guanine at the indicated time points. (B) Time-kill assays of *S. aureus* USA300 with the GuaB inhibitor G6 at the concentration of 2×, 4×, 8× MIC. Viable cells were serially diluted and plated on Mueller-Hinton agar plates after exposure to G6. The averages and standard deviations of three independent experiments are shown. The dotted line indicates the minimum limit of detection, 200 CFU/ml.

Figure S3. Hierarchical clustering of gene expression after treatment with G6 or ciprofloxacin. Each column represents a gene and each row represents a sample. Three replicates are shown for each treatment. The unsupervised clustering was generated with Partek Flow using log2 fold changes, average linkage as cluster distance metric, and Euclidean as point distance metric.

Figure S4. Changes in gene expression mapped to KEGG pathways. The log2 fold changes are indicated by color from -3 (green) to +3 (red).

Figure S5. Upregulation of molecular chaperone genes doesn't result in resistance to ciprofloxacin in either species. (A) Transcriptional changes of five major molecular chaperone genes in response to ciprofloxacin. RNA-Seq data with significance ($p < 0.05$) were marked with *. RNA-Seq data were validated with RT-qPCR. The averages and standard deviations of three independent experiments are shown. (B) Heat shock does not result in resistance to ciprofloxacin in either species. Ab19606 and MG1655 Δ*tolC* were grown to log phase at 37°C and shifted to 44°C for 30 min prior to addition of ciprofloxacin at 4× MIC. Viable cells were serially diluted and plated on M9 agar plate after exposure to ciprofloxacin.

Figure S6. GuaB inhibition leads to opposite transcriptional changes in genes required for tryptophan, glutamate and histidine biosynthesis in *A. baumannii* and *E. coli*. RNA-Seq data with significance ($p < 0.05$) were marked with *.

Synthesis of GuaB inhibitors.

Synthesis of G1, (2S)-N-(6-Chloro-3-pyridyl)-2-(2,4-dihydro-1H-pyrano[3,4-c]quinolin-9 ylamino)propenamide, and G2, (2S)-N-[4-Chloro-3-(dimethylamino)phenyl]-2-[[3-(hydroxymethyl)-6-quinolyl]amino]propenamide. Details were described previously (1).

Synthesis of G3, (2S)-N-(6-bromo-3-pyridyl)-2-[[3-(hydroxymethyl)-6-quinolyl]amino]propenamide. **G3** was made in a similar fashion as G2. ^{1}H NMR (400 MHz, DMSO-d6) δ 10.49 (s, 1H), 8.63 (dd, J = 2.8, 0.6 Hz, 1H), 8.46 (d, J = 2.0 Hz, 1H), 8.03 (dd, J = 8.7, 2.8 Hz, 1H), 7.82 (dd, J = 2.1, 1.0 Hz, 1H), 7.72 (d, J = 9.2 Hz, 1H), 7.58 (dd, J = 8.7, 0.6 Hz, 1H), 7.26 (dd, J = 9.1, 2.6 Hz, 1H), 6.65 (d, J = 2.6 Hz, 1H), 6.51 (d, J = 7.2 Hz, 1H), 5.33 – 5.25 (m, 1H), 4.60 (d, J = 4.4 Hz, 2H), 4.18 (p, J = 6.9 Hz, 1H), 1.49 (d, J = 6.9 Hz, 3H). LCMS M/Z (M+H) 401.

Synthesis of G4, (S)-4-(5-(2-((4-(1H-1,2,4-triazol-1-yl)phenyl)amino)propanamido)benzo[d]thiazol-2-yl)pyridine 1-oxide.

Scheme:

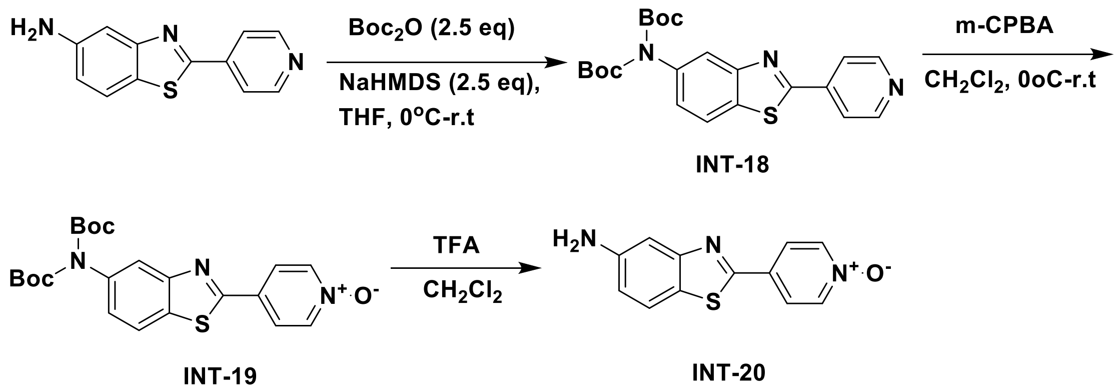

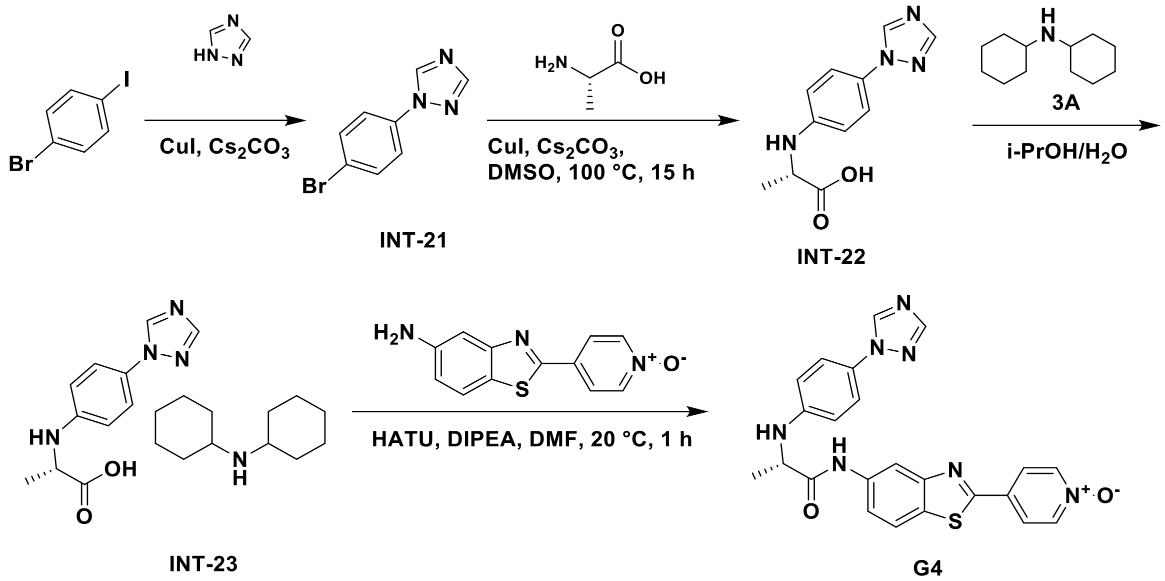

A solution of sodium bis(trimethylsilyl)amide (11.0 mL, 11.0 mmol) (1 M in THF) was added to a stirred solution of 2-(4-pyridyl)-1,3-benzothiazol-5-amine (1.00 g, 4.40 mmol) and di-tert-butyldicarbonate (2.40 g, 11.0 mmol) in anhydrous THF (60 mL) at 0°C under N_2. The resulting mixture was stirred at 20 °C for 1h. Saturated aqueous NH_4Cl was added to quench the reaction and the mixture was extracted with ethyl acetate. The combined organic phases were washed with brine, dried over Na_2SO_4, filtered and concentrated under reduced pressure. The residue was purified by flash column chromatography (0-40% ethyl acetate in petroleum ether) to yield tert-butyl (**INT-18,** *tert*-butyl *N*-*tert*-butoxycarbonyl-*N*-[2-(4-pyridyl)-1, 3-benzothiazol- 5-yl]carbamate (1.72 g, 91.4% yield) as a light-yellow solid. ^{1}H NMR (400 MHz, DMSO-d6) δ 8.81 (d, *J* = 4.8 Hz, 2H), 8.24 (d, *J* = 9.2 Hz, 1H), 8.08 - 7.98 (m, 3H), 7.41 (d, *J* = 8.4 Hz, 1H), 1.40 (s, 18H).

To a mixture of *tert*-butyl *N*-*tert*-butoxycarbonyl-*N*-[2-(4-pyridyl)-1, 3-benzothiazol- 5-yl]carbamate (**INT-18,** 8.0 g, 18.71 mmol) in dichloromethane (100 mL) was added portionwise 3-chloroperoxybenzoicacid (5.7 g, 28.1 mmol) at 0°C. After addition was complete, the mixture was warmed to 20°C and stirred for 4h. The mixture was quenched with saturated Na_2SO_3 solution. The organic layer was separated, washed with saturated brine solution, dried over Na_2SO_4 and concentrated under reduced pressure. The crude product was then purified by flash chromatography (10% MeOH in CH_2Cl_2) to give 4-(5-(bis(*tert*-butoxycarbonyl)amino)benzo[d]thiazol-2-yl)pyridine 1-oxide (**INT-19,** 6 g, 72.3% yield) as a

yellow solid. ^{1}H NMR (400MHz, $CDCl_3$) δ 8.31 (d, *J*=7.2 Hz, 2H), 7.97 (d, *J*=7.2 Hz, 2H), 7.92-7.84 (m, 2H), 7.27 (br d, *J*=2.0 Hz, 1H), 1.42 (s, 18H). LCMS m/z $[M+H]^+$ 444.0.

To a mixture of 4-(5-(bis(*tert*-butoxycarbonyl)amino)benzo[d]thiazol-2-yl)pyridine 1-oxide (15.0 g, 33.82 mmol) in dichloromethane (200 mL) was added trifluoroacetic acid (50 mL, 673.13 mmol) at 0°C. The mixture was stirred at room temperature for 3h. The mixture was concentrated under reduced pressure to give the crude product as a TFA salt. The residue was then suspended in ethyl acetate (100 mL) and treated with $NH_3.H_2O$ (30 mL). The mixture was then concentrated under reduced pressure, and purified by flash chromatography (10% MeOH in dichloromethane) to afford the free base desired product 2-(1-oxidopyridin-1-ium-4-yl)-1,3-benzothiazol- 5-amine (**INT-20,** 7 g, 85.1% yield) as a yellow solid. ^{1}H NMR (400MHz, DMSO-d_6) δ = 8.30 (d, =7.2 Hz, 2H), 7.98 (d, *J*=7.2 Hz, 2H), 7.72 (d, J=8.4 Hz, 1H), 7.14 (d, *J*=2.0 Hz, 1H), 6.79 (dd, *J*=2.2, 8.4 Hz, 1H), 5.40 (br, s, 2H).

To a solution of 1-bromo-4-iodobenzene (30 g, 106 mmol) in DMF (300 mL) was added Cs_2CO_3 (69 g, 212 mmol), copper(I) Iodide (6 g, 32 mmol) and 1,2,4-triazole (7.3 g, 106 mmol) at 15 °C. The reaction mixture was then stirred at 120°C for 16 h under N_2. After cooling to room temperature, the reaction mixtrue was concentrated to dryness and purified by silica gel chromatography (0-20%EtOAc in petroleum ether) to afford 1-(4-bromophenyl)-1,2,4-triazole (**INT-21,** 19 g, 80%) as a yellow solid. LCMS M/Z (M+H) 223.

To a solution of 1-(4-bromophenyl)-1,2,4-triazole (**INT-21**, 10 g, 44.63 mmol) in DMSO (100 mL) was added L-alanine (4.8 g, 53.88 mmol), Cs_2CO_3 (22 g, 67.52 mmol) and copper(I) Iodide (1.5 g, 7.88 mmol) at 10 °C. The reaction mixture was then placed under nitrogen atmosphere and stirred at 100°C for 15 h. After cooling to room temperature, the reaction mixture was purified by silica gel chromatography (0 - 10% methyl alcohol in dichloromethane then 100% methyl alcohol) to afford a partially purified crude product, which was suspened in water (300 mL), and acidified with 2 M aqueous HCl (19 mL). The resulting suspension was filtered. The filter cake was washed successively with water (30 mL x 3) and petroleum ether (30 mL x 3) to afford 2-[4-(1,2,4-triazol-1-yl)anilino]propanoic acid (**INT-22,** 8.5 g, 82%) as a white solid with 53% ee. ^{1}H NMR (400 MHz, DMSO-d_6) δ 12.66 (s, 1H), 9.00 (s, 1H), 8.12 (s, 1H), 7.50 (d, 2H), 6.67 (d, 2H), 6.24 (s, 1H), 4.08 - 3.94 (m, 1H), 1.39 (d, 3H). LCMS M/Z (M+H) 233.

To a solution of 2-[4-(1,2,4-triazol-1-yl)anilino]propanoic acid (**INT-22,** 2 g, 8.61 mmol) in 2-propanol (20 mL) and water (2 mL) was added dicyclohexylamine (1.6 g, 8.61 mmol) at 15°C and stirred at 15°C for 30 min. The reaction mixtrue was then filtered through celite and the filtrate was stirred at 0°C for 12 h. The precipitate formed was collected, washed with small amount of 2-propanol and dried. The filtrate was slightly concentrated, cooled to 0°C, seeded with a few crystals from 1st crop, stirred at 0°C for 1h to provide a 2nd crop. The process was repeated one more time. Combined yield of the (*S*)-2-((4-(1*H*-1,2,4-triazol-1-yl)phenyl)amino)propanoate dicyclohexylamine salt **INT-23** was (2 g, 56%) as a white solid with 99% ee. LCMS M/Z (M+H) 233.

To a solution of (*S*)-2-((4-(1*H*-1,2,4-triazol-1-yl)phenyl)amino)propanoate dicyclohexylamine salt (**INT-23,** 2 g, 4.84 mmol) and HATU (2.2 g, 5.8 mmol) in DMF (15 mL) was added a solution of 2-(1-oxidopyridin-1-ium-4-yl)-1,3-benzothiazol-5-amine (1.7 g, 4.84 mmol) and DIPEA (4.3 mL, 24.18 mmol) in DMF (5 mL) at 0°C. The mixture was then stirred at 20°C for 30 min and purified by silica gel chromatography (0 - 5% methyl alcohol in dichloromethane) to afford the title compound (725 mg, 33%) as a yellow solid with 98% ee. ^{1}H NMR (400 MHz, CD_3OD) δ 8.84 (s, 1H), 8.48 (d, 1H), 8.42 (d, 2H), 8.17 (d, 2H), 8.08 (s, 1H), 7.97 (d, 1H), 7.63 (dd, 1H), 7.54 (d, 2H), 6.82 (d, 2H), 4.11 (q, 1H), 1.61 (d, 3H), LCMS M/Z (M+H) 458.

Synthesis of G5, (2*S*)-2-(4-pyrazol-1-ylanilino)-*N*-[2-(4-pyridyl)-1,3-benzothiazol-5-yl]propenamide. G5 was prepared in a similar fashion to **G4.** ^{1}H NMR (400 MHz, CD_3OD) δ = 8.74 - 7.82 (m, 2H), 8.50 (d, J = 2.0 Hz, 1H), 8.09 - 8.07 (m, 2H), 8.99 - 7.97 (m, 2H), 7.66 - 7.62 (m, 2H), 7.46 (d, J = 8.8 Hz, 2H), 6.80 (d, J = 8.8 Hz, 2H), 6.45 - 6.43 (m, 1H), 4.09 (q, J = 7.2 Hz, 1H), 1.60 (d, J = 7.2 Hz, 3H).
LCMS $[M+H]^+$ = 441.1.

Synthesis of G6, (*S*)-*N*-(4-chloro-3-morpholinophenyl)-2-((3-(hydroxymethyl)quinolin-6-yl)amino)propenamide. G6 was made in a similar fashion to **G2.** ^{1}H NMR (400MHz, MeOD-d_4) δ 8.53 (s, 1H), 8.22 - 7.78 (m, 1H), 7.61 (s, 1H), 7.48 (s, 1H), 7.34 - 7.16 (m, 2H), 6.94 - 6.81 (m, 1H), 6.80 - 6.24 (m, 1H), 4.86 - 4.61 (m, 3H), 4.30 - 4.16 (m, 1H), 3.86 - 3.79 (m, 5H), 3.60 - 3.46 (m, 1H), 3.02 - 2.97 (m, 4H), 1.71 - 1.61 (m, 9H). LCMS m/z (M+H): 525.

Synthesis of G7, (S)-2-((4-(1H-pyrazol-5-yl)phenyl)amino)-N-(2-(pyridin-4-yl)benzo[d]thiazol-5-yl)propanamide

Scheme:

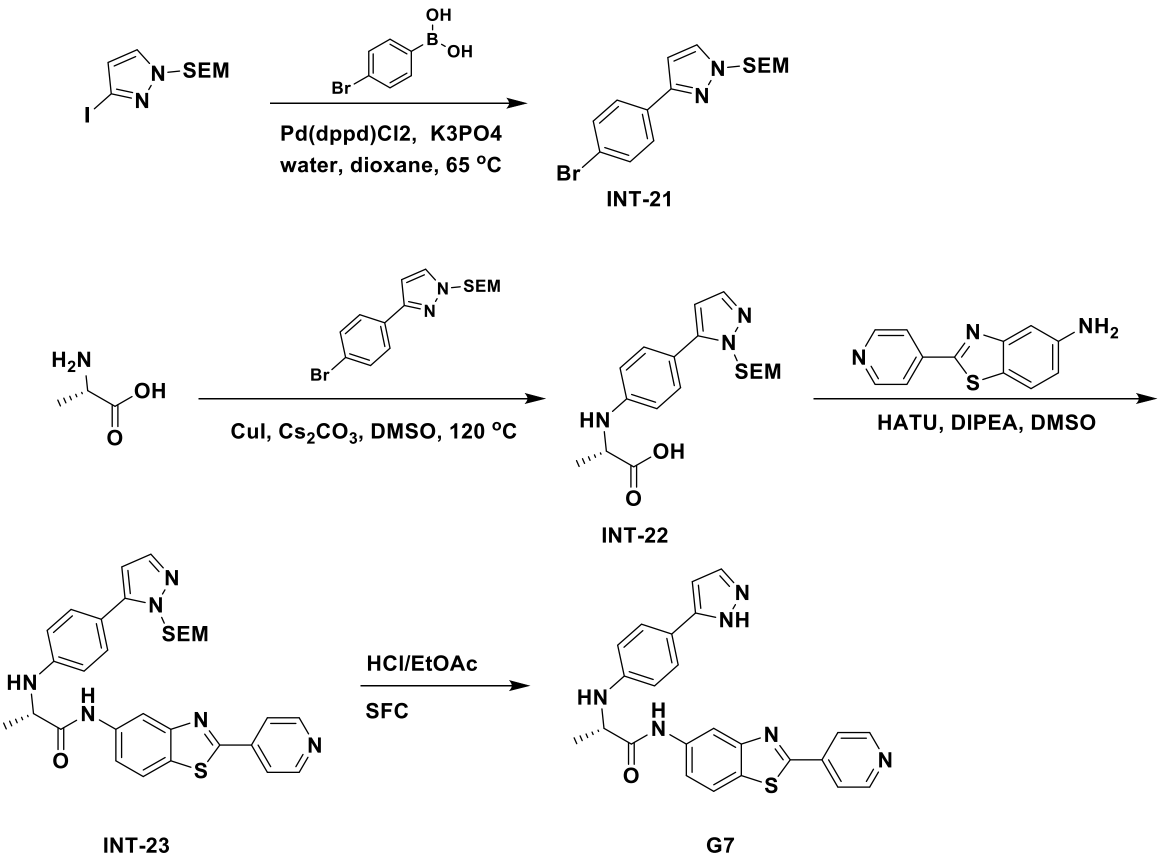

A solution of 2-[(4-iodopyrazol-1-yl)methoxy]ethyl-trimethyl-silane (10.0 g, 30.8 mmol), (4-bromophenyl)boronic acid (6.8 g, 33.9 mmol), Pd(dppd)Cl2 (1.1g, 1.5 mmol) and formylperoxypotassium; potassium hydride (19.7 g, 92.5 mmol) in dioxane/H2O (200 mL/20 mL) was stirred at 65 °C for 3 h under N2. The reaction mixture was poured into H2O (500 mL) and extracted with EtOAc (500 mL x 2). The combined organic layers were washed with brine (600 mL), dried over anhydrous Na2SO4, filtered and the filtrate was concentrated under reduced pressure. The residue was purified by flash silica gel chromatography (ethyl acetate in petroleum ether from 0% to 6%) to give product 2-[[4-(4-bromophenyl)pyrazol-1-yl]methoxy]ethyl-trimethyl-silane (**INT-21**, 7.5g ,21.2 mmol, 68.8% yield) as an orange oil.

A mixture of *L*-alanine (0.45 g, 5.05 mmol), CuI (0.48 g, 2.53 mmol), cesium carbonate (4.9 g, 15.15 mmol) and 2-[[3-(4-bromophenyl)pyrazol-1-yl]methoxy]ethyl-trimethyl-silane

(**INT-21,** 1.8 g, 5.05 mmol) in DMSO (5 mL) was stirred at 120°C for 16h under N_2. After cooling, the reaction mixture was diluted with water (20 mL), then adjusted the pH ~5 with aqueous HCl (2 M) and extracted with EtOAc (200 mL). The organic layer was dried and concentrated to afford (2*S*)-2-[4-[2-(2-trimethylsilylethoxymethyl)pyrazol-3-yl]anilino]propanoic acid (**INT-22**, 2 g, 68%) as black oil, used directly in the next step without further purification. LCMS M/Z: [M+H] 362.

To a solution of (2*S*)-2-[4-[2-(2-trimethylsilylethoxymethyl)pyrazol-3-yl]anilino]propanoic acid (**INT-22**, 2.0 g, 3.43 mmol) in DMSO (3 mL) was added DIPEA (2.83 mL, 17.15 mmol), 2-(4-pyridyl)-1,3-benzothiazol-5-amine (0.78 g, 3.43 mmol) and HATU (2.6 g, 6.86 mmol) at 20°C. The mixture was then stirred at 20°C for 16h, diluted with EtOAc (50 mL) and washed with sat. NaCl (10 mL x 5). The organic layer was dried and concentrated. The crude residue was purified by flash chromatography on silica gel eluting with 0 - 30% EtOAc in petroleum ether to afford (S)-N-(2-(pyridin-4-yl)benzo[d]thiazol-5-yl)-2-((4-(1-((2-(trimethylsilyl)ethoxy)methyl)-1H-pyrazol-5-yl)phenyl)amino)propanamide (**INT-23**, 0.8 g, 41%) as a yellow solid. LCMS M/Z: [M+H] 571.

To a mixture of (2*S*)-*N*-[2-(4-pyridyl)-1,3-benzothiazol-5-yl]-2-[4-[2-(2-trimethylsilylethoxy-methyl)pyrazol-3-yl]anilino]propanamide (**INT-23**, 0.8 g, 1.4 mmol) in THF (1 mL) was added a solution of 4 N HCl in EtOAc (4 mL, 16 mmol). The mixture was stirred at 25°C for 16h, then poured into sat. $NaHCO_3$ (50 mL) and extracted with EtOAc (200 mL). The organic layer was dried and concentrated. The crude residue was purified by reverse phase HPLC (acetonitrile 30 - 60/10 mM NH_4HCO_3 in water) to afford the title compound as a yellow solid. LCMS M/Z: [M+H] 441. SFC (Chiralcel OJ-3 50 x 4.6mm I.D., 3um) showed 57.7 % ee. The compound was further purified by SFC (OJ(250mm x 30mm,10um)), supercritical CO_2/EtOH (0.1% NH3.H_2O) and the major peak (RT = 5.301 min) was collected to afford (S)-2-((4-(1H-pyrazol-5-yl)phenyl)amino)-N-(2-(pyridin-4-yl)benzo[d]thiazol-5-yl)propanamide (**G5,** 12 mg, 12%) as a white solid. ^{1}H NMR (400MHz, DMSO-d_6) δ 13.21 - 12.10 (m, 1H), 10.45 (s, 1H), 8.78 (d, 2H), 8.56 (s, 1H), 8.12 (d, 1H), 8.01 (d, 2H), 7.69 (d, 1H), 7.57 - 7.48 (m, 3H), 6.68 (d, 2H), 6.46 (s, 1H), 6.20 (d, 1H), 4.14 (t, 1H), 1.47 (d, 3H). LCMS M/Z: [M+H] 441.

Table S1. Additional information on IC_{50} of GuaB inhibitors (μM)

Compound	GuaB[Ab]				GuaB[Ec]				GuaB[Sa]			
	n	mean	sem	cv	n	mean	sem	cv	n	mean	sem	cv
G1	3	2.71×10^{-4}	4.55×10^{-5}	16.8	1	4.78×10^{-1}	NA	NA	4	3.84×10^{-4}	4.16×10^{-5}	10.8
G2	4	6.66×10^{-4}	1.17×10^{-4}	17.6	1	1.58×10^{-2}	NA	NA	3	3.50×10^{-3}	5.01×10^{-4}	14.3
G3	2	9.75×10^{-4}	4.16×10^{-4}	42.6	2	9.10×10^{-2}	5.73×10^{-3}	6.30	2	2.45×10^{-3}	1.11×10^{-3}	45.3
G4	2	6.17×10^{-4}	2.90×10^{-5}	4.70	3	9.03×10^{-1}	1.10×10^{-1}	12.2	3	6.20×10^{-4}	1.09×10^{-4}	17.5
G5	2	3.94×10^{-4}	1.13×10^{-4}	28.7	2	5.69×10^{-1}	1.30×10^{-2}	2.28	2	5.11×10^{-4}	3.35×10^{-5}	6.56
G6	2	5.64×10^{-4}	7.25×10^{-5}	12.9	2	5.90×10^{-3}	2.51×10^{-4}	4.26	2	8.01×10^{-4}	1.30×10^{-4}	16.2
G7	2	8.24×10^{-4}	5.77×10^{-4}	70.0	2	1.20	2.87×10^{-1}	23.8	2	3.94×10^{-4}	1.69×10^{-4}	42.8

Ab: *A. baumannii* ATCC19606; *Ec*: *E. coli* MG1655*; Sa: S. aureus* USA300.
n: number of measurements; mean: average of IC_{50}; sem: standard error of the mean; cv: coefficient of variation, cv= 100× sem/mean.

Table S5. Bacterial strains used in this study

Strain	Species	Genotype	Reference
Ab19606	*Acinetobacter baumannii*	Wild-type	ATCC 19606
Ab19606 *ΔguaB*	*Acinetobacter baumannii*	*ΔguaB::*Kan^R	(1)
Ab19606 *ΔlpxA*	*Acinetobacter baumannii*	*ΔlpxA::*Kan^R	(2)
Ab19606 *ΔadeJ*	*Acinetobacter baumannii*	*ΔadeJ::*Kan^R	(1)
MG1655	*Escherichia coli*	Wild-type	ATCC 700926
MG1655 *ΔguaB*	*Escherichia coli*	*ΔguaB::*Kan^R	This study
MG1655 *ΔtolC*	*Escherichia coli*	*ΔtolC::*Kan^R	(3)
MG1655 *ΔtolC ΔguaB*	*Escherichia coli*	*ΔtolC ΔguaB::*Kan^R	This study
USA300	*Staphylococcus aureus*	*ΔhsdR ΔsauUSI*	(4)
USA300 *ΔguaB*	*Staphylococcus aureus*	*ΔhsdR ΔsauUSI ΔguaB*	(5)

Table S6. qPCR primers used in this study

Gene	Forward primer	Reverse primer	Probe
Ab_16S	GAG TAT GGG AGA GGA TGG TAG A	CGT ACC TCA GCG TCA GTA TTA G	/56-FAM/AGG TGT AGC /ZEN/GGT GAA ATG CGT AGA /3IABkFQ/
Ab_dnaJ	GCA GAT CGT CAG CAA ACA TTA G	CAC GAA TAG CCT CGC CTT TA	/56-FAM/TGG ATA ATG /ZEN/GTG ACC GCG TTC GTT /3IABkFQ/
Ab_dnaK	CTG ACA TCT CTG ACG TCA TCT T	CGT CTT TAC GTG GCT CTC TAC	/56-FAM/TGG CAT ACG /ZEN/AGA CTG ACC ACC AAC /3IABkFQ/
Ab_groL	TTT ACG CCG TGC GAT TGA	CGA AGT TAC CCT CAC CAT TCT T	/56-FAM/CGT CAA ATC /ZEN/GTT GCG AAT GCT GGT /3IABkFQ/
Ab_groS	TGA GCA ACA TTC GTC CAT TAC A	GCA GCA GAA CCT GGC AAT A	/56-FAM/TAC GCG ACG /ZEN/AAT CAC AAC GCG ATC /3IABkFQ/
Ab_grpE	GCG TGA GTC TGA GAA GCA TAA A	ACC TTC AAG TAC AGG CGT TTC	/56-FAM/TTT AGA ACG /ZEN/CGC AAT TCA GGC AGC /3IABkFQ/
Ab_entA	CCT GTT CGG TAC TGG CTT AAA	TCA CCT GGA TCT ACT CTC ACT C	/56-FAM/TGC AGG TGG /ZEN/AGG TGC ATT ATC TGT /3IABkFQ/
Ab_entB	GTA TCT TCA CGG TGT GGT CAA	CTC TAG CCA AGG TCG TGT AAT G	/56-FAM/TCT GTA GTG /ZEN/AGG CAA GTG GCA ACA /3IABkFQ/
Ab_entC	TTT GTG GCT CAC CAA CTC A	GTC ACT ACC CAC TCA CCA TTA C	/56-FAM/TTA GTC GGC /ZEN/TGG ATG GAT GCG G/3IABkFQ/
Ab_entD	GCT AAC AGC TTT CGG GAT AGA	CTA ATT GAC GAA TGA AGC GTG AG	/56-FAM/AGG TCC AGC /ZEN/TCA GCC AAA TCA AAG A/3IABkFQ/
Ab_entE	ATG ACT CCG ACG AGC AAA TC	CCT TCT GGG ACT TCC CTA TAC T	/56-FAM/CGA CAC AAG /ZEN/GGC GTC CTA TCA GTT /3IABkFQ/
Ab_entF	GAA GTA CAG GGA CTC CGA AAT G	ACG GTG TTA CCG CCA TAA TC	/56-FAM/AAA CAC GCT /ZEN/ACA ACA GAC GAT CTC GG/3IABkFQ/
Ec_16S	GTC AGC TCG TGT TGT GAA ATG	CCC ACC TTC CTC CAG TTT ATC	/56-FAM/ACG AGC GCA /ZEN/ACC CTT ATC CTT TGT /3IABkFQ/
Ec_dnaJ	GTG AAG GCA ACA ACC TGT ATT G	CAA GGG TCG GTA CTT CGA TTT	/56-FAM/AGT CCC GAT/ZEN/CAA CTT CGC TAT GGC/3IABkFQ/
Ec_dnaK	GTA TCG CTG GTC TGG AAG TAA A	CAG GTC ATA AAC CGC GAT AGT	/56-FAM/TTA CGG TCT/ZEN/GGA CAA AGG CAC TGG/3IABkFQ/
Ec_groL	TGG ACG TGG TTG AAG GTA TG	GAA CGG GCT TTC CAG TTC TA	/56-FAM/ACC GTG GCT/ZEN/ACC TGT CTC CTT ACT/3IABkFQ/
Ec_groS	GAA AGT TGG CGA CAT CGT TAT TT	TTC GGA CAT GAT CAA CAC TTC T	/56-FAM/ACG ATG GCT/ZEN/ACG GTG TGA AAT CTG AG/3IABkFQ/
Ec_grpE	CGC TGG AAG TGG CTG ATA AA	GCC AAA CTT ACG CAC AAC ATC	/56-FAM/AAC CCG GAT/ZEN/ATG TCT GCG ATG GTT/3IABkFQ/
Ec_entA	GCG TTC ACT CAG GAG CAA TA	CTA ACA GTC GCT GAC ACA CTT	/56-FAM/TCG GCA ACA /ZEN/TCC ATC ACT TCG GTC /3IABkFQ/
Ec_entB	AAT TAC TGC CAG CAC CTA TCC	ATC CAG ACC GTA GTC GAT CA	/56-FAM/CCG ATG AAC /ZEN/CGT TCG ATG ACG ACA /3IABkFQ/
Ec_entC	CAG GCG ATG AAA GAG GTA CTG	TCA AAG GGA GTT GCG AGA TG	/56-FAM/ACA CGT TCC /ZEN/TTC TTC TCC ACA GCT /3IABkFQ/
Ec_entD	CTA TAA ATG TGT GCC CGC AAT C	GAC GAG ATA CCA CGG CTA ATG	/56-FAM/CGG CAG TAT /ZEN/TAG CCA CTG TGG GAC /3IABkFQ/
Ec_entE	GCG AGC GAC AGT TGA GTT AT	GCG GTT TCA CCA GGT TTA ATG	/56-FAM/AAA CTA CAC /ZEN/GCG AGG TTA TCC GCC /3IABkFQ/
Ec_entF	TGT GCA GCG TAC CGA TTT AG	CCC AGT CGA CGC ATA AAG ATA A	/56-FAM/TGT GCA ATC /ZEN/GTA TGG ACT ACG CCG /3IABkFQ/

Table S7. Structure data collection and refinement statistics.

	GuaB^{Ab} G6 (9C4M)
Wavelength	1.105
Resolution range	72.56 - 2.48 (2.569 - 2.48)
Space group	P 1 21 1
Unit cell	103.157 126.992 127.381 90 102.959 90
Total reflections	385994 (39280)
Unique reflections	113072 (11270)
Multiplicity	3.4 (3.5)
Completeness (%)	99.79 (99.90)
Mean I/sigma(I)	6.88 (2.18)
Wilson B-factor	22.60
R-merge	0.173 (0.5893)
R-meas	0.2053 (0.6961)
R-pim	0.1095 (0.3675)
CC1/2	0.981 (0.757)
CC*	0.995 (0.928)
Reflections used in refinement	113056 (11269)
Reflections used for R-free	5766 (583)
R-work	0.1865 (0.2590)
R-free	0.2275 (0.3260)
CC (work)	0.949 (0.867)
CC (free)	0.918 (0.759)
Number of non-hydrogen atoms	21854
macromolecules	19652
ligands	736
solvent	1770
Protein residues	2680
RMS(bonds)	0.102
RMS(angles)	2.88
Ramachandran favored (%)	97.95
Ramachandran allowed (%)	2.01
Ramachandran outliers (%)	0.04
Rotamer outliers (%)	2.92
Clashscore	2.46
Average B-factor	29.04
macromolecules	28.67
ligands	23.69
solvent	34.43

Statistics for the highest-resolution shell are shown in parentheses.

References

1. Kofoed EM, Aliagas I, Crawford T, Mao J, Harris SF, Xu M, Wang S, Wu P, Ma F, Clark K, Sims J, Xu Y, Peng Y, Skippington E, Reeder J, Ubhayakar S, Baumgardner M, Yan Z, Chen J, Park S, Zhang H, Yen C-W, Lorenzo M, Skelton N, Liang X, Chen L, Hoag B, Li CS, Liu Z, Wai J, Liu X, Liang J, Tan M-W. 2024. Discovery of GuaB inhibitors with efficacy against Acinetobacter baumannii infection. mBio.

2. Tiku V, Kew C, Kofoed EM, Peng Y, Dikic I, Tan M-W. 2022. Acinetobacter baumannii Secretes a Bioactive Lipid That Triggers Inflammatory Signaling and Cell Death. Front Microbiol 13:870101.

3. Alexander MK, Miu A, Oh A, Reichelt M, Ho H, Chalouni C, Labadie S, Wang L, Liang J, Nickerson NN, Hu H, Yu L, Du M, Yan D, Park S, Kim J, Xu M, Sellers BD, Purkey HE, Skelton NJ, Koehler MFT, Payandeh J, Verma V, Xu Y, Koth CM, Nishiyama M. 2018. Disrupting Gram-Negative Bacterial Outer Membrane Biosynthesis through Inhibition of the Lipopolysaccharide Transporter MsbA. Antimicrob Agents Ch 62:e01142-18.

4. Monk IR, Shah IM, Xu M, Tan M-W, Foster TJ. 2012. Transforming the Untransformable: Application of Direct Transformation To Manipulate Genetically Staphylococcus aureus and Staphylococcus epidermidis. mBio 3:e00277-11.

5. Kofoed EM, Yan D, Katakam AK, Reichelt M, Lin B, Kim J, Park S, Date SV, Monk IR, Xu M, Austin CD, Maurer T, Tan M-W. 2016. De Novo Guanine Biosynthesis but Not the Riboswitch-Regulated Purine Salvage Pathway Is Required for Staphylococcus aureus Infection In Vivo. J Bacteriol 198:2001–2015.
